# Piperazine skeleton in the structural modification of natural products: a review

**DOI:** 10.1080/14756366.2021.1931861

**Published:** 2021-06-03

**Authors:** Run-Hui Zhang, Hong-Yan Guo, Hao Deng, Jinzi Li, Zhe-Shan Quan

**Affiliations:** a Affiliated Hospital of Yanbian University, Yanji, Jilin, China; b College of Pharmacy, Yanbian University, Yanji, Jilin, 133002, China

**Keywords:** Piperazine, natural product, pharmacological activity, structure–activity relationship

## Abstract

Piperazine moiety is a cyclic molecule containing two nitrogen atoms in positions 1 and 4, as well as four carbon atoms. Piperazine is one of the most sought heterocyclics for the development of new drug candidates with a wide range of applications. Over 100 molecules with a broad range of bioactivities, including antitumor, antibacterial, anti-inflammatory, antioxidant, and other activities, were reviewed. This article reviewed investigations regarding piperazine groups for the modification of natural product derivatives in the last decade, highlighting parameters that affect their biological activity.

## 1. Introduction

The piperazine ring, an important class of N-heterocyclic bioactive natural products, is extensively present in biologically active compounds^1^. The piperazine scaffold has been recognised as a privileged structure in drug discovery and is widely distributed in biologically active compounds employed in several different therapeutic fields, including antitumor^2^, antibacterial^3^, anti-inflammatory^4^, antipsychotic^5^^,^^6^, anti-Alzheimer^7^^,^^8^, antifungal^9^, and anti-diabetics^10^. The N-4 nitrogen of piperazine can be used as a basic amine, while the N-1 nitrogen can easily introduce hydrogen bond acceptors and hydrophobic groups through other heterocyclics without necessitating the addition of a stereocenter^11^^,^^12^.

Piperazine core has two primary nitrogen atoms which exert the improvement in pharmacokinetic features of drug candidates because of their appropriate pKa^13^. These nitrogen sites lead to the essential increase in water solubility of the drug-like molecules thereby playing a crucial role in the bioavailability. Maintaining a balance between pharmacodynamic and pharmacokinetic profiles of drug-like molecules is the important factor in designing and developing of new drugs, thus, one of the goals in the drug discovery process is to design molecules with a high affinity for their targets and appropriate physicochemical properties. For this purpose, the characteristics of the piperazine template make this molecular subunit a useful and well positioned system in the rationale design of drugs^14^.

Natural products are closely related to human development. Through the process of metabolic evolution, bacteria, fungi, and plants have co-evolved along with animals, greatly exploring the chemical space, and producing a large number of natural products that can alter the senses and behaviour of animals^15^. Designing hybrid molecules based on natural products has become an effective strategy, including combining two or more biologically active compounds from natural sources, and is expected to deliver these compounds to target organs simultaneously. Reportedly, this combination strategy has lower toxicity when compared with traditional single-target molecule co-administration methods. Additionally, hybrid molecules improved dose compliance, reduced drug interactions, and the cost of preclinical evaluation^16^^,^^17^. Natural products have always been an alternative source of chemical substances for exploring biological systems, as well as biological pre-verification prototypes for chemical probes and drug discovery, which are considered sources of new drug research and development owing to their unique chemical diversity^18^^,^^19^. A significant number of compounds have been developed from plants, microbial metabolites, and marine organisms. Between 1981 and 2019, approximately 23.5% of the new drugs approved globally, 33.6% of the approved small molecule drugs, and 75.6% of the drugs in the anti-infective field were derived from natural products and their derivatives^20^. Given the importance and significant biological activity of the piperazine skeleton in natural products, in recent years, an increasing number of scientific articles, books, and patents have been based on piperazine ring modifications of natural products. A growing wealth of literature reflects the research interests and importance of this field, as well as the potential for developing drugs based on piperazine groups^21–23^. Therefore, this article combined the latest literature reports, especially from the perspective of biological activity, to introduce the research progress in this field. We anticipate that this review will provide novel concepts and research directions for the modification of natural products.

## 2. Biological effects of natural products containing the piperazinyl moiety

### 2.1. Antitumour activity

**Figure 1.**
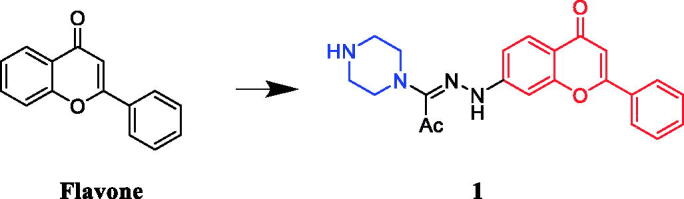
Chemical structures of flavone and its derivative.

A series of N1-(flavon-7-yl)amidrazones incorporating N-piperazines were synthesised by Abu-Aisheh^24^. Structure-activity relationships (SARs) shows that the presence of the phenyl group at C-2 does make a difference for the anti-K562 activity whereas none of the compounds having a methyl substituent at C-2 showed any activity against the K562 cells lines, more compounds of the flavone series (where there is a phenyl group linked to C-2) and only a few of the 2-methyl series displayed activity, and leaving the piperazine ring unsubstituted is better for the anti-K562 activity while for breast cancer, the anti-T47D activity resembles that of the K562 activity. Among them, compound **1** (Figure 1) was reportedly a potential antineoplastic agent against T47D cells (breast cancer cell line), with an IC_50_ value of 1.42 µM. Unfortunately, its activity is not as effective as the positive control drug doxorubicin (IC_50_ = 0.33 µM).

**Figure 2.**
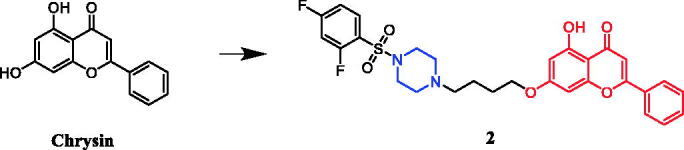
Chemical structures of chrysin and its derivative.

Anticancer properties of chrysin-based sulfonylpiperazines were reported by Pecere et al.^25^ SARs show that the presence of halogen atom(s) was found essential to get better potency against SK-OV-3, HeLa, and A‐549 cell lines. Derivative **2** (Figure 2) with dual fluorine atom substitution on the sulfonylpiperazine ring exhibited the best activity against SK-OV-3 with least 12.67 ± 0.74 μg/ml of IC_50_ and was observed as potent as gefitinib (IC_50_ = 12.31 ± 0.33 μg/ml). Furthermore, in case of inhibition of HeLa cell line, again derivative with 2,4-difluoro substituent demonstrated remarkable 4.67 ± 1.42 μg/mL of IC_50_, which was better than gefitinib (IC_50_ = 17.92 ± 1.50 μg/ml). Patel et al. documented the synthesis of chrysin-piperazine conjugates and evaluated the *in vitro* anticancer activity against HeLa, CaSki, and SKOV-3 cancer cell lines. The findings revealed that the nature and position of the functional groups on the piperazine core may contribute to the expected antioxidant and anticancer effects from the perspective of structure-activity^26^.

**Figure 3.**
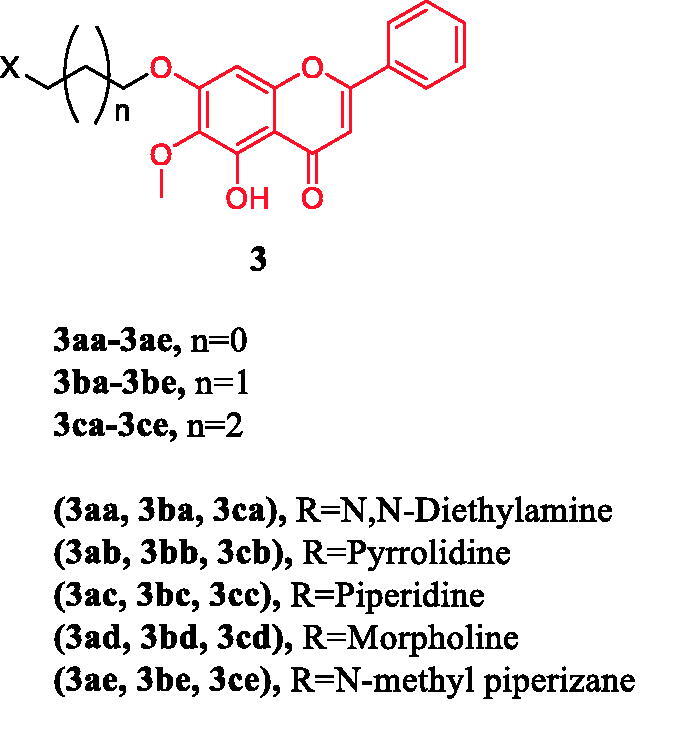
Chemical structures of oroxylin A and its derivatives.

Oroxylin A (5,7-dihydroxy-6-methoxyflavone) is a naturally occurring monoflavonoid isolated from the root of Scutellaria baicalensis Georgi and exhibits potent anticancer activities *in vitro* and in vivo. Fu et al. synthesised three series of oroxylin derivatives by connecting a nitrogen-containing hydrophilic, heterocyclic ring to the C7-OH via a varying length of the carbon chain. Compounds (**3ca–3cc**) (Figure 3) containing a 4-carbon spacer were particularly potent with respect to cellular inhibition of all the three cell lines, with IC_50_ value ranging from 1.42 to 9.52 µM, approximately 5- to 20-fold more potent than oroxylin A, except for **3cd** and **3ce**. Compound **3cb** and **3cc** were found to be the most potent, with IC_50_ of 1.42 µM (HCT116) and 2.98 µM (HepG2), respectively. The results indicate that the length of the chain between the terminal heterocyclic substitutes and oroxylin A is an important factor for their potency. However, with a morpholinyl or N-methyl piperazinyl group at the terminal of 7-O-alkyl oroxylin A, compound **3cd** and **3ce** (with IC_50_ at 12.5–50.6 µM) displayed moderate levels of inhibition, which was similar to that of oroxylin A but over 4–25-fold (HepG2), 2–30-fold (HCT116), 3–6-fold (BCG823) less potent than **3ca**–**3cc**. The morpholinyl or N-methyl piperazinyl substitution in the compounds **3ad**–**3ae** (with IC_50_ at 15.4–67.6 µM) and **3bd**–**3be** (with IC_50_ at 30.4–74.8 µM) also exhibited lower activities than substitutions with N, N-diethylamino (**3aa** and **3ba**), pyrrolyl (**3ab** and **3bb**) or a piperidyl (**3ac** and **3bc**) group, respectively, regardless to the length of the carbon chain. It may be attributed to the volume of the side chain. Annexin V/PI staining experiments proved that compound **3cc** has the highest *in vitro* efficacy on HepG2 cells among the derivatives. The difference of **3cc** between the inhibition of cell proliferation and the apoptotic induction indicated that **3cc** was more likely to be a necrosis-inducing agent or both apoptosis/necrosis inducer. Thus, the novel 7-O-alkylamino derivative of oroxylin A, compound **3cc**, could be a promising antitumor candidate, and further *in vitro* and in vivo biological character evaluations are warranted^27^.

**Figure 4.**
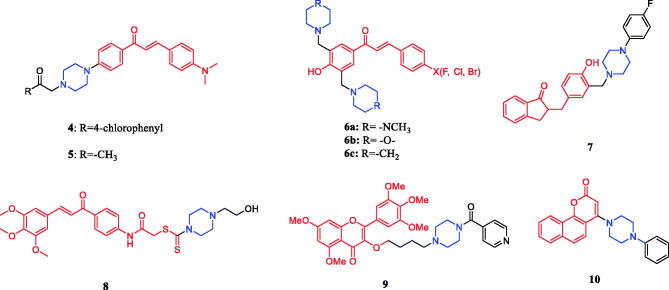
Chemical structures of chalcone and its derivatives.

Novel hybrid compound **4** (Figure 4) between chalcone and piperazine demonstrated potent antitumor activity against A549 (a human lung cancer cell line), Hela and SGC7901 cells (IC_50_ = 5.24, 0.19, and 0.41 µM, respectively), which was better than positive control drug cisplatin (IC_50_ = 11.54 20.52, and 12.44 µM, respectively)^28^. In addition, chalcone-piperazine derivative **5** (Figure 4) exhibited inhibitory activity against the A549 cell line, with an IC_50_ value of 0.19 µM, which was better than positive control drug cisplatin (IC_50_ = 11.54 µM)^29^. Mannich bases **6a, 6b, 6c** (Figure 4) were derivatives of chalcone. Though compounds showed a low inhibition potency towards hCA I (25–43%) and hCA II (6–25%) isoforms at an inhibitor concentration of 10 µM, they were more selective (1.5–5.2 times) towards hCA I isoenzyme. Compared with piperidine and N-methylpiperazine, Mannich bases **6b** containing morpholine (substituted by fluorine, chlorine or bromine on the benzene ring) had the highest average SI value. The experimental results showed that the chalcone-piperazine hybrid compounds containing acetophenone had good anti-tumour activity. Among them, the substitution of halogen atoms on the benzene ring had a great impact on the anti-tumour activity, and the compounds substituted by fluorine atoms on the benzene ring showed the best activity^30^. A series of chalcone-piperazine derivatives as mono Mannich bases were designed and synthesised by Tugrak and co-workers. The amine part was altered to N-phenylpiperazine, N-benzylpiperazine, 1–(2-fluorophenyl)piperazine, 1–(4-fluorophenyl) piperazine, and 1–(2-methoxyphenyl)piperazine. All synthesised compounds synthesised demonstrated a good inhibition profile towards hCA I and II isoenzymes, with K*_i_* values between 29.6 and 58.4 nM and 38.1–69.7 nM, respectively. Additionally, compound **7** (Figure 4) revealed the highest tumour selectivity value (TS: 59.4), possibly by inducing necrotic cell death. The research showed cytotoxicity of some compounds containing piperazine in chalcone-Mannich base derivatives is stronger than other compounds containing different secondary amines (such as morpholine, piperidine, pyrrolidine, dimethylamine, diethylamine, dipropylamine, dibenzylamine)^31^^,^^32^. Novel chalcone-dithiocarbamate hybrids were designed, synthesised, and evaluated for their antiproliferative activity against select cancer cell lines (MGC803, MCF7, and PC3). Investigations evaluating modifications and structure-activity relationship (SAR) revealed that substituents on the piperazine unit are important for their inhibitory activity. Among these analogs, **8** showed the best inhibitory activity against PC3 cells (IC_50_ = 1.05 µM), which was better than positive control drug 5-FU (IC_50_ = 29.31 µM). Cellular mechanism studies elucidated that **8** (Figure 4) could inhibit colony formation, arrest the cell cycle at the G2/M phase, and induce DNA damage against PC3 cells. On replacing the piperazine ring with the morpholine or pyrrolidine group, the activity showed a particularly noticeable decrease^33^. The discovery of new chromen-4-one derivatives as telomerase inhibitors has been reported. Compound **9** (Figure 4) showed potent inhibitory activity against telomerase. Compound **9** exhibited high activity against Hela, SMMC-7721, SGC-7901, U87 and HepG2 cell lines with IC_50_s of 1.02, 1.33, 1.35, 2.50 and 4.12 µM, respectively, compared to the positive doxorubicin (IC_50_ = 0.21, 0.81, 0.98, 2.24, and 2.87 µM, respectively). The preliminary SARs showed that most compounds had good activity against SGC-7901 cells, but, almost all compounds possessed poor activity against HepG2 cells. And the substituent R showed a large effect for the activity, heterocycle is better than that benzene ring (compound **9**). Further, the substituent number of benzene rings showed great influence on anticancer. Preliminary investigations revealed that compound **9** inhibited telomerase activity by decreasing the expression of dyskerin. Furthermore, the docking results showed that the target compound could strongly interact with residues LYS189 and Asp254 after introducing the piperazinphenyl methanone structure. In vivo experiments, compound **9** could evidently abate pathological changes of hepatic lobules in DEN-induced rats. The hepatic cells showed markedly reduced atypia, full cytoplasm and high differentiation^34^. Reportedly, 4-amino- 2H-benzo[h] chromen-2-one analogs were synthesised and evaluated for their anticancer activity. Compound **10** (Figure 4) displayed potent androgen receptor binding affinity, as well as strong cytotoxic activity against LNCaP-hr cells (IC_50_ = 0.52 ± 0.11 µM), which was better than positive control drug finasteride (IC_50_ = 14.5 µM). The SAR results showed that the 4-position substituent of the piperazine ring and the ortho-substituted phenyl analogs had relatively good inhibitory activity^35^.

**Figure 5.**
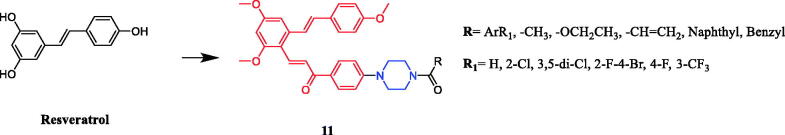
Chemical structures of resveratrol and its derivatives.

Novel resveratrol-chalcone-piperazine amide derivative **11** (R=–CH = CH_2_) (Figure 5) showed superior cytotoxic activity against A549 and HeLa cells (IC_50_ = 0.26 and 7.35 µmol/L, respectively), which was better than positive control drug cisplatin (IC_50_ = 11.54 and 20.52 µmol/L, respectively). And fluorescence-activated cell sorter (FACS) analysis showed that the compound effectively induced apoptosis in A549 cells. The structure of the compound had a significant effect on anticancer activity. The preliminary structure-activity relationship shows that the anticancer activity was related to the number and category of substituents on the benzene ring, but had nothing to do with the position of substituents, and the lack of electronic benzene ring led to positive activities^36^.

**Figure 6.**
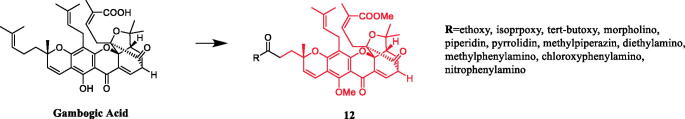
Chemical structures of gambogic acid and its derivative.

A gambogic acid derivative **12** (Figure 6) containing a methylpiperazine substituent was found to possess a most potent inhibitory activity against A549 and BGC-823 cells among all compounds tested. In A549 cell line, compound **12** exhibited the highest potency with an IC_50_ value of 0.12 µM, which was 10-fold more potent than gambogic acid. In BGC-823 cell line, compound **12** displayed the best potency with an IC_50_ value of 0.57 µM, which was almost 5-fold more potent than gambogic acid. The SARs at the C-37 site of gambogic acid shows that the removal of hydrophobic prenyl tail and introduction of hydrophilic carboxyl group slightly reduced the anti-tumour activity. Further introduction of hydrophobic motifs such as methyl, isoprenyl and tert-butyl groups had little effect on improving the potency. Inspiringly, substituted by aliphatic amines showed obviously increased activities. Meanwhile, the aliphatic amines also could improve the solubility and drug-like properties of the derivatives. Notably, compound **12** containing methylpiperazine substituents was the most excellent derivative with the IC_50_ values ranging from 0.12 to 3.10 µM against the five cell lines. Phenylamine groups were not a good moiety for improving the activity at the C-37 site. In addition, although compounds with the flexible amino acid side chains ought to have greatly improved solubility, they were found to be less active or even mostly inactive against all the five cell lines. The introduction of glycine group to C-37 site could increase the selectivity against HCT-116 and HepG2 cells. This study found that compounds with aliphatic amines had higher anti-tumour activity than aniline, improving the solubility and drug-like properties of derivatives^37^.

**Figure 7.**
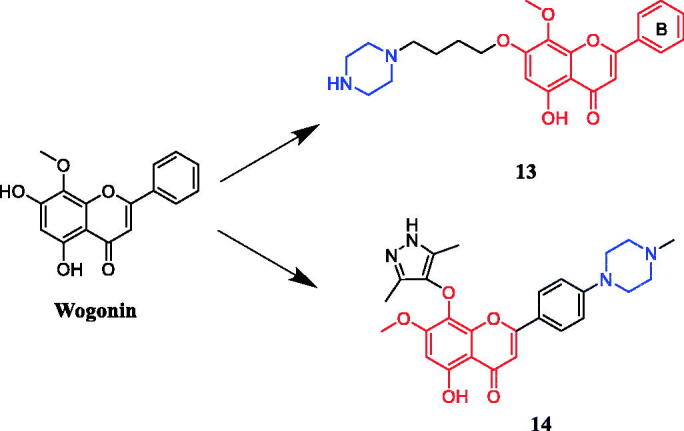
Chemical structures of wogonin and its derivative.

Li and co-workers revealed that wogonin-piperazine derivative **13** (Figure 7) possessed the highest potency against HepG2, A549, and BCG-823, with IC_50_ values of 1.07 µM, 1.74 µM, and 0.98 µM, respectively, which was better than positive control drug 5-FU (IC_50_ = 17.2, 16.1 and 10.1 µM, respectively) and parent compound wogonin (IC_50_ = 19.0, 15.8 and 16.5 µM, respectively). The results showed that the anti-tumour activity was enhanced when the 7-position of wogonin was occupied by aliphatic amines, in contrast, wogonin derivatives substituted at 8-position or B-ring were generally less potent than the control 5-fluorouracil^38^. In another study, wogonin derivative **14** (Figure 7) showed excellent activity against MV4-11 (leukaemia cell line) (IC_50_ = 20 nM) and CDK9 (IC_50_ = 19.9 nM) cell growth. In addition, compound **14** showed much improved physicochemical properties, such as water solubility, compared with the parent compound wogonin. The follow-up studies showed that the compound **14** is selective towards CDK9-overexpressing cancer cells over normal cells. Preliminary mechanism studies on the anticancer effect indicated that **14** inhibited the proliferation of MV4-11 cells via caspase-dependent apoptosis. In addition, highlighted compound **14** showed significant antitumor activity in mouse acute myeloid leukaemia (AML) models without producing apparent toxic effects in vivo. Additionally, among the synthesised compounds, the piperazine and methylpiperazine substituted derivatives exhibited higher antiproliferative potency against the HepG2 cell line, while morpholine and pyrrolidine substituted analogs showed dramatically lower antiproliferative activity^39^.

**Figure 8.**
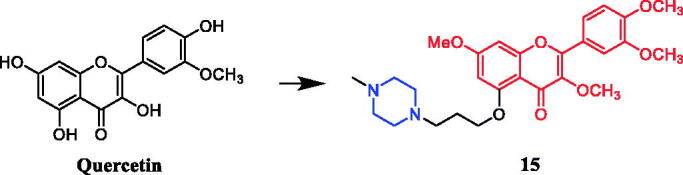
Chemical structures of quercetin and its derivative.

In an investigation targeting prostate cancer cells, 6 of the 48 quercetin derivatives synthesised contained piperazine moieties. Moreover, quercetin derivative **15** (Figure 8) demonstrated promising efficacy against the PC-3 cell line, with an IC_50_ value of 4.47 ± 0.92 µM, being over 2–13 times more potent than quercetin. The experimental results indicated that modification of the hydroxyl groups at positions 3 and 5 significantly improved their inhibitory activity against prostate cancer cells, besides, the 48 derivatives are generally more effective in inhibiting the proliferation of PC-3 and LNCaP cells than DU145 cells^40^.

**Figure 9.**
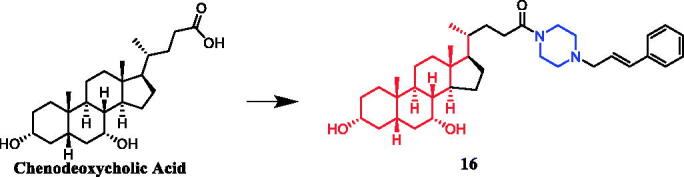
Chemical structures of chenodeoxycholic acid and its derivative.

A series of novel bile acid (including ursodeoxycholic acid, cholic acid, chenodeoxycholic acid) hybrid compounds comprising piperazine were designed for anticancer activity. Among them, compound **16** (Figure 9) as the most potent against KMS-11, a multiple myeloma cell line, with an LD_50_ value of 8.5 ± 0.5 µM. Concerning the structure–activity relationship, the best activity was obtained with compound **16** having cinnamylpiperazinyl group in the side chain and hydroxyl group at C-7 of the steroid skeleton. These results prompt us in a future study to incorporate other amino-substituted cinnamylpiperazinyl groups in the side chain of chenodeoxycholic acid^41^^,^^42^.

**Figure 10.**
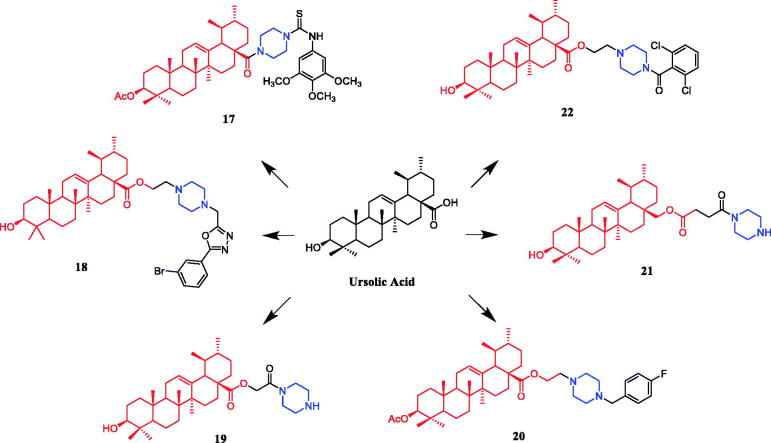
Chemical structures of ursolic acid and its derivative.

**Figure 11.**
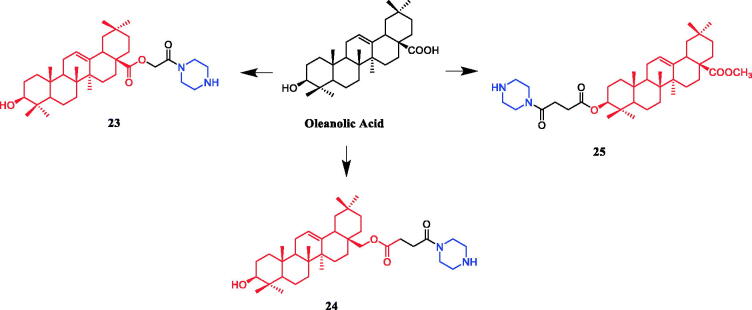
Chemical structures of oleanolic acid and its derivative.

**Figure 12.**
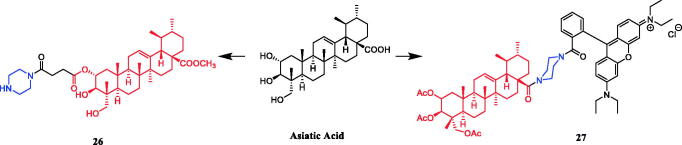
Chemical structures of asiatic acid and its derivatives.

Currently, an ursolic acid (UA) derivative, obtained from the leaves and berries of many natural plants, is under development as a lead anticancer agent, which has also been modified by introducing piperazine rings to develop novel compounds. In 2015, Shao and co-workers revealed that substituted piperazine coupled to UA could induce cancer cell apoptosis and arrest cell cycle at the G0/G1 stage in HT-29, HepG2, and RL95-2 cells^43^. In the same year, UA derivative **17** (Figure 10) with piperazine was reported as a potential anticancer agent, inducing cell apoptosis by G1 cell cycle arrest. Compound **17** displayed the best proliferation among this series of compounds, with IC_50_ values of 9.82 µM, 18.97 µM, 13.64 µM, 5.40 µM and 11.06 µM against MGC-803, HCT-116, T24, HepG2 and A549 cancer cell lines, respectively. And compound **17** showed better potent antiproliferative activity than UA (as well as 5-FU) against these five cancer lines, with IC_50_ values of 27.08 µM, 38.78 µM, 29.29 µM, 30.21 µM and 35.79 µM. SARs indicated that the synchronous introduction of piperazine and thiourea at C-28 and an acyl group at C-3 may improve the antitumor activity of UA, and solely introduction of an acyl piperazine thiourea at C-28 may decrease the antitumor activity. Moreover, it could be concluded that para-substituents in the phenyl ring were important than meta-substituents to their antiproliferative activities. Western blot and qRT-PCR (quantitative real-time PCR) experiments demonstrated that compound **17** may induce apoptosis through both intrinsic and extrinsic apoptosis pathways. Moreover, **17** has shown a pronounced increase in Fas and caspase-8 activity. Therefore, these studies indicated that the target compound may induce apoptosis, arrest cell cycle progression at the S phase and increase the activity of caspase-3 to inhibit cell growth. It is worth noting that piperazine was used as a linker in this UA modification^44^. UA derivatives targeting hypoxia-inducible factor-1α (HIF-1α) by introducing a 1,3,4-oxadiazole moiety, triazolone, and piperazine ring were designed and synthesised. Compound **18** (Figure 10) demonstrated excellent activity inhibiting the expression of HIF-1α, with IC_50_ = 38.1 µM, possessing a lower appreciable cytotoxic activity (IC_50_ > 100 µmol/L) than that of UA (IC_50_ = 23.8 µmol/L). SAR indicating that the triazolone moiety was favourable for HIF-1α inhibition in the UA derivatives, and introducing a piperazine ring to the oxadiazole structure can also enhance the biological activity of the compounds^45^. In 2016, Tian et al. reported that the introduction of a piperazine moiety at the C-28 position of oleanolic acid (OA) and UA demonstrated a superior growth inhibition against MCF-7 cancer cells than the lead compounds or positive control gefitinib, including derivatives **19** (Figure 10)**, 21** (Figure 10)**, 23** (Figure 11) and **24** (Figure 11), with IC_50_ values of 40.27 ± 4.88 µM, 56.85 ± 4.88 µM, 12.1 µM, and 14.2 µM, respectively^46^^,^^47^. In 2019, some UA derivatives containing a piperazine moiety presented significant antitumor activities against the cancer cell lines evaluated. The structure-activity relationship studies revealed that the introduction of thiazole on A ring and triazole or tetrazole moiety on C-28 has little help to improve antitumor activities. The introduction of piperazine or homopiperazine can significantly improve the antitumor activity. And 4-fluorobenzyl and piperazine moieties are the crucial anticancer functional groups of these compounds. Among them, compound **20** (Figure 10) was found most effective, demonstrating IC_50_ values of 2.6 µM and 2.1 µM against the HeLa and MKN45 cell lines, respectively. And compound **20** was better than positive control drug cisplatin (IC_50_ = 15.1 and 2.8 µM, respectively) and was better than parent compound UA (IC_50_ = 15.1 and 16.7 µM, respectively). Compound **20** decreased the apoptosis regulator (BCL2/BAX) ratio, which subsequently disrupted the mitochondrial potential and induced apoptosis and significantly suppressed the growth of Hela xenografts in nude mice^48^. In 2012, UA derivative **22** (Figure 10) was described as a potent antitumor agent that induced cell apoptosis and inhibited growth against MGC-803 cells (gastric cancer cell), with an IC_50_ value of 2.50 ± 0.25 µM, which was better than positive control drug hydroxycamptothecin (IC_50_ > 20 µM) and parent compound UA (IC_50_ = 24.32 ± 0.57 µM). The SAR studies revealed that incorporation of an acyl piperazine moiety at C-28 while retaining the polar group at C-3 significantly improved the antitumor bioactivities of the compounds^49^. In 2015, Zhao et al. synthesised and investigated a series of piperazine-containing pentacyclic triterpene derivatives. OA derivative **25** (Figure 11) and asiatic acid derivative **26** (Figure 12) was reported as the most promising antitumor agents, with IC_50_ values in the range of 7.05–13.13 µM, when evaluated against MCF-7, HeLa, and A549 cell lines. The pronounced influence of the incorporation of piperazine on antiproliferative effects in the series of synthesised compounds could be explained on the capacity for the formation of hydrogen bonds, improvement of water-solubility, and adjustment of molecular physicochemical properties. Furthermore, **25** and **26** can induce cell cycle arrest in all of the three cells^50^.

Asiatic acid piperazine derivative **27** (Figure 12) shows distinct cytotoxicity for several human tumour cell lines, e.g. EC_50_ (A2780) = 8 ± 2 nM, which was better than parent compound asiatic acid (IC_50_ = 28.2 µM). Interestingly, compound **27** showed non-linear, bimodal dose-response relationships against two human tumour cell lines (HT29 and 518A2)^51^.

**Figure 13.**
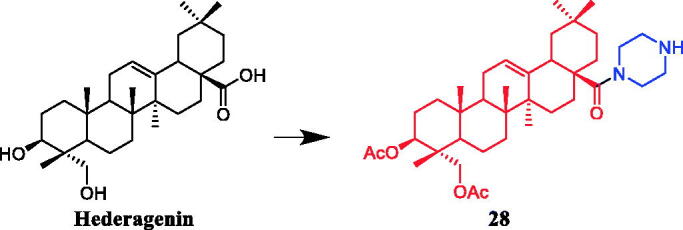
Chemical structures of hederagenin and its derivatives.

A series of C-28 amide derivatives of hederagenin were synthesised, with or without acetyl groups at the 3 and 23 positions of ring A, to develop effective cytotoxic agents. The amide derivatives were cytotoxic for a variety of human tumour cell lines. In general, the hydroxylated derivatives were less active than the acetylated derivatives. Among them, compound **28** (Figure 13) carrying a piperazinyl presented good cytotoxic activity against A2780 cells (EC_50_ = 1.9 µM), which was better than parent compound hederagenin (IC_50_ > 30 µM)^52^.

**Figure 14.**
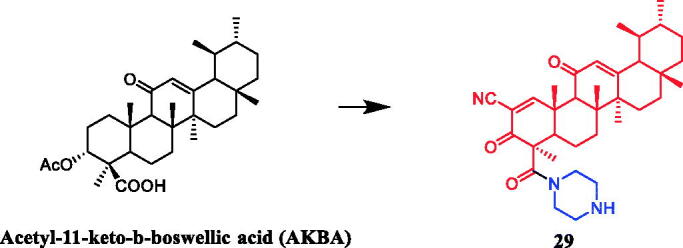
The structure of pentacyclic triterpenoid AKBA.

Li et al. synthesised a series of acetyl-11-keto-β-boswellic acid (AKBA) derivatives and evaluated their anticancer activity. Indicating that the cytotoxic activity of AKBA was improved effectively through modification of ring A and C-24. Introduction of 2-cyano-3-oxo-1-en system in ring A of boswellic acids increased cytotoxicity. The esterification of free carboxyl acid at C-24 resulted in a sharp decrease in the activity. AKBA derivative **29** (Figure 14) with 2-cyano-3-oxo-1-en in ring A and piperazine amide at C-24 was reported as the most active in inhibiting the growth of prostate cancer PC-3 (IC_50_ = 0.04 µM) and LNCaP (IC_50_ = 0.27 µM) cell lines, with 796-fold and 102-fold more potent than AKBA. The mechanism of action of **29** indicated that cell cycle arrest and apoptosis induction were related to its anti-proliferative effects. **29** could regulate the expression levels of cell cycle- and apoptosis-related proteins, such as p21, cyclin D1, Mcl-1 and NOXA, in a concentration-dependent manner. AKBA had a weak ability to inhibit Pin1 activity, which was improved by the modification on ring A^53^.

**Figure 15.**
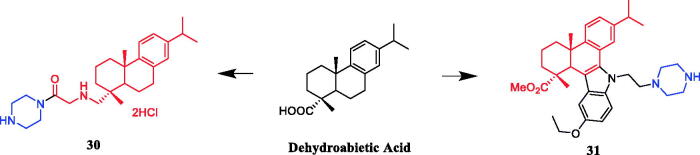
Chemical structures of dehydroabietic acid and its derivatives.

Zhao and co-workers designed and synthesised several series of dehydroabietic acid piperazine derivatives. The results indicated that compound **30** (Figure 15) with piperazine moiety showed selectivity for HepG-2 over MCF-7, with IC_50_ values of 23.56 and 62.55 µM, respectively^54^. In another study, 1H-dibenzo[a, c]carbazole derivatives of dehydroabietic acid bearing different N-(piperazin-1-yl)alkyl side chains were designed, synthesised, and evaluated for their *in vitro* anticancer activities against three human hepatocarcinoma cell lines. SAR indicated that methyl, methoxyl, ethoxyl and chloro groups anchored on the indole moiety were more beneficial to the anticancer activity. In addition, the derivatives containing 12-Me and 12-OEt generally showed greater cytotoxic activities than their analogs with the same substituents at C-10, while compound with 10-OMe substituent was relatively more active than compound with 12-OMe. Especially, Compound **31** (Figure 15) with 12-OEt substituent showed strong cytotoxic activity against SMMC-7721, HepG2, and Hep3B cell lines, with IC_50_ values of 1.39 ± 0.13, 0.51 ± 0.09, and 0.73 ± 0.08 µM, respectively. Compared with lead compound and the positive control doxorubicin, it exhibited considerably more potent anticancer activities against three cancer cells and lower cytotoxicity to normal hepatocyte cell line QSG-7701 (IC_50_: 12.52 ± 0.58 µM). Compound **31** could significantly inhibit MEK1 kinase activity with IC_50_ of 0.11 ± 0.02 µM. Furthermore, compound **31** increased the level of intracellular reactive oxygen species (ROS), reduced the mitochondrial membrane potential, destroyed the integrity of the cell membrane, ultimately resulting in the proliferation and apoptosis of HepG2 cells. Therefore, it could be a potent MEK inhibitor and a potential anticancer drug^55^.

**Figure 16.**
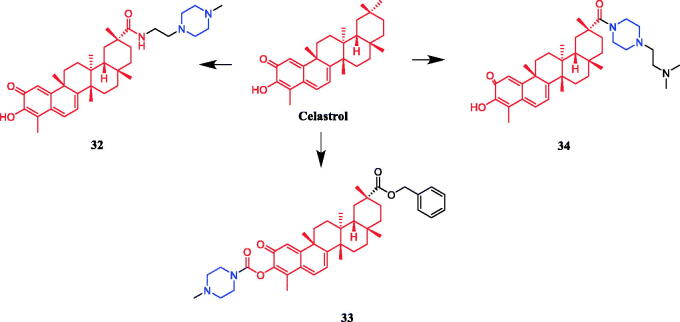
Chemical structures of celastrol and its derivatives.

To explore the SAR improve drug-like properties of celastrol derivatives, 23 derivatives targeting the Hsp90-Cdc37 interaction were synthesised by Jiang et al. SAR indicated that the derivatives that incorporated polar groups on the C29 carboxyl showed promising Hsp90–Cdc37 disruption activities and compound **32** (Figure 16) containing the piperazine moiety exhibited the most significant Hsp90-Cdc37 activity disruption, with an IC_50_ value of 4.71 ± 0.14 µM, which was better than parent compound celastrol (IC_50_ = 6.49 ± 0.27 µM). Compound **32** improved solubility and permeability. Compound **32** also exhibited potent antitumor activities, with IC_50_ values of 1.01–1.12 µM against MCF-7, Panc-1 and A549, better than parent compound celastrol (IC_50_ = 2.02–2.48 µM). Compound **32** could induce the degradation of clients Akt and Cdk4 dose-dependently, suggesting that Compound **32** exerted their antitumor activities through Hsp90 inhibition in part. Moreover, Compound **32** induced cell cycle arrest at G0/G1 phase and physiological apoptosis in Panc-1 cells. Compound **32**, with improved Hsp90–Cdc37 disruption activity, cytotoxicity and good druglike^56^. A series of 3-carbamate and 29-ester celastrol derivatives were synthesised by Shan et al. SAR indicated that the activity of derivatives with piperazine was better than that with aniline, Piperidine and morpholine. The substitution of piperazine and morpholine by piperidine and aniline weakened the activity, which might indicate that nitrogen atom and oxygen atom here are favourable as hydrogen bond acceptor but the alkyl substituents and hydrophobic groups not. The nitrogen atom is more suitable than the oxygen atom here and the groups on it make difference to the activity. The activity gets lower when the substituents get bigger. Meanwhile, we observed a significant loss of cytotoxicity when the size of the substituent group on C-3 hydroxyl become as big as 8–10 carbons length. However, when the size was smaller than that, it did not cause an obvious change in the activity. It indicated a threshold about the size of this position, which is located in front of C-3 hydroxyl. It might affect the activity and pharmacokinetic property of celastrol, and the stereo structure here may improve the selectivity. The carbamates were introduced nitrogen and oxygen atoms as new hydrogen bond acceptors and hydrophilic centre. The antitumor activity of compound **33** (Figure 16) against several cancer cell lines, with IC_50_ values of 5.18 and 1.02 µmol/L against A549 and Bel7402 cell lines. It is slightly less active than the lead compound celastrol (IC_50_ = 2.12 µmol/L and 0.44 µmol/L), but compound **33** revealed a dose-dependent inhibition and better inhibitory activity, with 70.96% inhibition of A549 cells at a dose of 12 mg/kg when compared with celastrol at 12 mg/kg (*p* < .05) *in vivo*^57^. Piperazine-containing celastrol derivative **34** (Figure 16) was revealed a potential telomerase inhibitor against the MGC-803 cell line, presenting an IC_50_ value of 1.00 ± 0.17 µM, which was better than positive control drug 5-FU (IC_50_ = 3.58 ± 0.40 µM), AMD (IC_50_ = 1.19 ± 0.07 µM) and parent compound celastrol (IC_50_ = 1.55 ± 0.25 µM)^58^. In 2017, a series of triterpenoic acid derivatives containing the piperazine moiety exhibited low EC_50_ values against several human tumour cell lines^59^.

**Figure 17.**
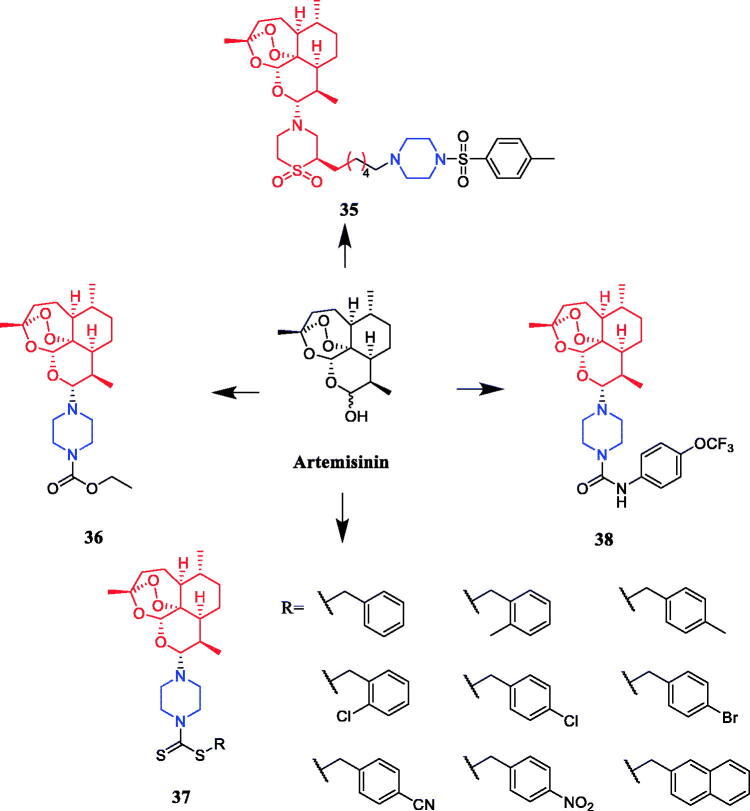
Chemical structures of artemisinin and its derivatives.

Reportedly, several artemisone-piperazine-sulphonamide derivatives have been synthesised. The antiproliferative activity of the new compounds was evaluated against human hepatoma cells. Compound **35** (Figure 17) showed activity against SMMC-7721 cell lines (IC_50_ = 0.09 µmol/mL), which was better than parent compound artemisone (IC_50_ = 0.44 µmol/mL)^60^. In 2017, a series of novel artemisinin derivatives were designed and synthesised by Sun et al. and were evaluated using the MTT assay against ten cell lines. These derivatives were generally more effective in inhibiting cancer cell growth than artemisinin (IC_50_ > 40 µM). Among them, compound **36** (Figure 17) which bore an N-ethoxycarbonyl-piperazine group was the most active against HepG2 and PLC-PRF-5 cells (the IC_50_ values were 4.1 and 5.7 µM, respectively) and presented no cytotoxicity on L-02 cells, and **36** might be a valuable potential antitumor drug against HCC. SAR found that introducing the piperazine group into the C10 position of artemisinin could enhance the antiproliferative effect on certain cancer cells to some degree. The structure of compounds **36**, which showed the highest activity against hepatocellular carcinoma cells, inspired to improve the oil solubility of the piperazine group into the C10 position. Furthermore, this substrate could induce cell cycle arrest in the G2/M phase and apoptosis in HepG2 cells^61^. Reportedly, Yu et al. synthesised twelve artemisinin-piperazine- dithiocarbamate derivatives, all of which had stronger *in vitro* antitumor activity than dihydroartemisinin. In particular, artemisinin derivative **37** (R = 4-cyanobenzyl) (Figure 17) showed promising inhibitory activity against the SMMC-7721 cell line, with an IC_50_ value of 0.0025 ± 0.04 µM, which was better than positive control drug vincristine (IC_50_ = 0.0103 ± 0.03 µM), cytosine arabinoside (IC_50_ = 0.0271 ± 0.04 µM) and dihydroartemisinin (IC_50_ ≫ 0.1000 µM) and lower toxicity against LO2 cell lines, presenting an IC_50_ of 0.18 ± 0.04 µM^62^. In another study, artemisinin derivatives containing fluorine atoms synthesised by Li et al. were evaluated for their antiproliferative potencies against U87MG, SHSY5Y, MCF-7, MDA-MB-231, A549, and A375 cancer cell lines. Compound **38** (Figure 17), the most active derivative containing the piperazine moiety, presented an IC_50_ value of 2.1 µM against the human breast cancer MCF-7 cells by inducing apoptosis and G1-phase arrest and showed lower cytotoxicity (IC_50_ = 6.7 µM) than doxorubicin (IC_50_ = 0.9 µM) against L02. SAR showed that the structural modification of artemisinin, i.e. introducing the group of piperazine with attached amide bond into C10 position of artemisinin, led to a dramatic increase of the inhibitory effect on certain cancer cells to some degree^63^.

**Figure 18.**
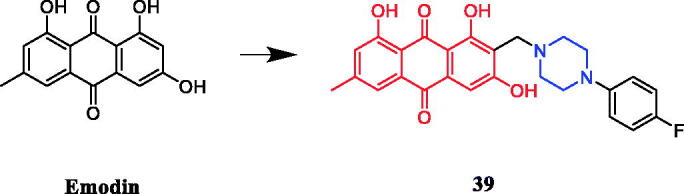
Chemical structures of emodin and its derivatives.

Narender et al. generated a variety of emodin derivatives and found that the piperazine-containing derivative **39** (Figure 18) strongly inhibited the proliferation of HepG2 and MDA-MB-231 cancer cell line with an IC_50_ of 10.44 µM and 5.027 µM, respectively, which is comparable to marketed drug epirubicin (IC_50_ of 4.6 µM and 7.7 µM, respectively). SAR indicated that O-alkylation and C-alkylation of emodin improve the activity in both cell lines, whereas esterification only improves the activity in the MDA-MB-231 cell line. The derivatives **39** was capable of arresting the cell cycle at the G1/S phase, demonstrating caspase-dependent apoptosis in the HepG2 cell line, as well as DNA intercalating activity^64^.

**Figure 19.**
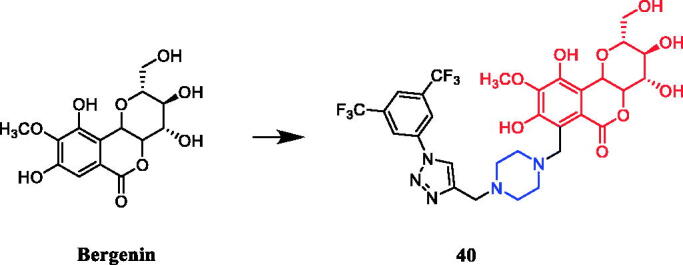
Chemical structures of bergenin and its derivatives.

A series of bergenin-1,2,3-triazole hybrids were synthesised and evaluated for their potent activity against cancer cell lines. Among them, piperazine-containing compound **40** (Figure 19) exhibited potent activity against HeLa and A-549 cells, with IC_50_ values of 1.33 µM and 1.86 µM, respectively, and was reportedly equipotent to doxorubicin (IC_50_ values 1.98 µM and 1.34 µM respectively). Preliminary structure-activity relationship indicates that the presence of a substituent such as a chlorine atom or a cyano, floro and CF_3_ group on aromatic triazole partner enhanced activity with IC_50_ values ranging from 1.33 to 9.9 µM on A459 cell as well as HeLa cell lines within the studied series. In addition, flow cytometry analysis had demonstrated that compound **40** triggered cell cycle arrest in the G2/M phase and induced apoptosis in a dose and time-dependent manner. Taken together, compound **40** effectively inhibited tubulin polymerisation, disrupted intracellular tubulin-microtubule balance, resulting in prolonged G2/M cell cycle arrest. Docking studies also indicated a strong hydrophobic interaction with tubulin, thus leading to stable binding, consequently leading to apoptosis of cancer cells^65^.

**Figure 20.**
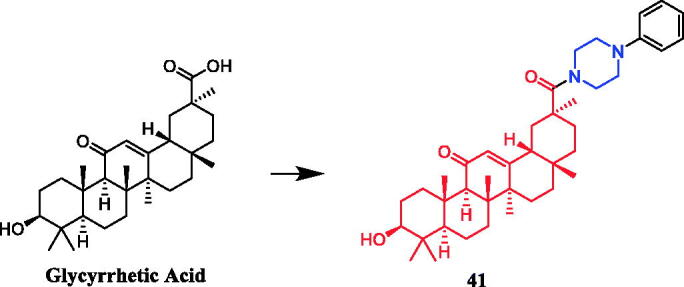
Chemical structures of glycyrrhetic acid and its derivatives.

Yan et al. revealed that glycyrrhetic acid-piperazine derivative **41** (Figure 20) exhibited the best inhibitory activity against MCF-7 cells, with an IC_50_ value of 1.08 µM, which was better than the positive control vandetanib (IC_50_ = 1.40 µM). SAR can be concluded that a fragment of a phenylpiperazine skeleton plays a significant role in the antiproliferative effect *in vitro*. Among the thirteen glycyrrhetic acid derivatives containing phenylpiperazine skeleton, compound **41**, which has smaller steric hindrance showed better inhibitory capability than the others, and compounds that contain alkylpiperazine had the same trend. Compound **41** also showed potent inhibitory activity against vascular endothelial growth factor receptor 2 (VEGFR2) tyrosine kinase, with an IC_50_ value of 0.35 µM. Docking simulations were performed to discover the binding mode, and the results indicated that compound **41** could bind well to VEGFR2 at the active site^66^.

**Figure 21.**
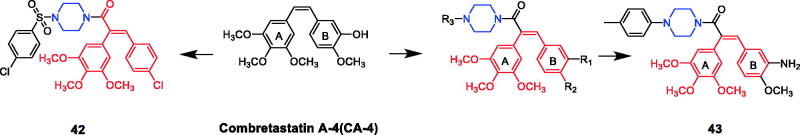
Chemical structures of combretastatin-A4 and its derivatives.

Combretastatin-A4 linked sulphonyl piperazine derivative **42** (Figure 21) exhibited remarkable cytotoxicity in all cancerous cell lines investigated. Compound **42** displayed an IC_50_ of 0.36 ± 0.02 µM against A549 cells, which was more active than the lead compound Combretastatin-A4 (IC_50_ = 0.43 ± 0.02 µM). Further investigations revealed that it could significantly inhibit tubulin assembly, effectively acting at the colchicine binding pocket of tubulin. The SARs of these derivatives indicated that the 4-chloro substitution on the benzene ring C4 of the sulphonamide showed effective activity, and the activity was significantly reduced when sulphonamide ring is substituted by other substituents such as hydrogen, methyl, methoxy and tert-butyl. In addition, the selectivity of compound **42** to A549 cells is almost 3 times that of normal HaCaT cells^67^. O’Boyle et al. reported the synthesis and anticancer activities of combretastatin-A4 piperazine conjugates. The SAR research pointed out that the impact on the viability of MCF-7 breast cancer cells was related to the substituents on the piperazine ring, and the general trend in potency was as follows: phenylpiperazine > acetylpiperazine ≈ cinnamylpiperazine > benzylpiperazine. Amino-substituted p-tolylpiperazine **43** (Figure 21) was the most potent of all piperazine derivatives, suggesting that an amino group is advantageous. Derivative **43** revealed an IC_50_ value of 83 nM against MCF-7 breast cancer cells, unfortunately, the activity of **43** is not as high as the lead compound combretastatin-A4 (IC_50_ = 3.9 nM). Furthermore, compound **43** was selectively toxic to MCF cells, inducing G2/M phase arrest and apoptosis of MCF-7 cells, but not in peripheral blood monocytes, and inducing cleavage of DNA repair enzyme poly adenosine diphosphate ribose polymerase (PARP) in MCF-7 cells^68^.

**Figure 22.**
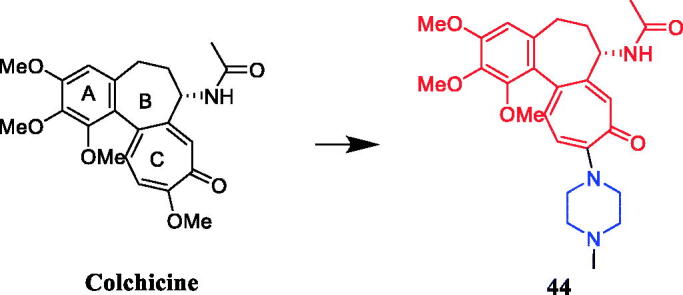
Chemical structures of colchicine and its derivatives.

Singh et al. reported the synthesis and anticancer activities of colchicine derivatives. SAR indicated that A- and C-rings of colchicine scaffold are the minimum structural features necessary for high-affinity drug–tubulin binding, and C-ring modifications and B-ring substitutions (particularly, on acetamido group) are well tolerated for their anti-tubulin activity. Compound **44** (Figure 22) showed notably lower P-glycoprotein (Pgp) induction activity when compared with colchicine, and good antiproliferative activity against the Colo-205 cell line, with an IC_50_ value of 1.0 ± 0.006 µM. Unfortunately, the activity of **44** is not as high as the lead compound colchicine (IC_50_ = 0.032 µM)^69^.

**Figure 23.**
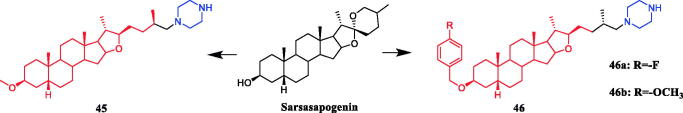
Chemical structures of sarsasapogenin and its derivatives.

Sarsasapogenin-piperazine derivatives **45**, **46a,** and **46 b** (Figure 23) displayed significant inhibitory effects against several tumour cell lines. Derivatives **45** revealing potent toxicity against the A549 cancer cell line, with an IC_50_ value of 1.70 µM, which was more active than positive control timosaponin A-III (IC_50_ = 11.92 µM) and 5-fluorouracil (IC_50_ = 42.15 µM). Compounds **46a** and **46b** exhibited significant cytotoxic activities against the six cell lines, being more potent than their parent compound sarsasapogenin. Furthermore, the *p*-fluorobenzyloxy series of compounds generally exhibited stronger cytotoxicities against all the tested cancer cells compared with the benzyloxy and p-methoxybenzyloxy series, and the substitution of pyrrolidinyl and piperazinyl groups at the C26 position was the preferred option for these compounds to display antitumor activities. Compound **46b** exhibited excellent cytotoxic activity against MCF-7 cell line (IC_50_ = 2.95 µM), and was 16.7-fold more potent than sarsasapogenin. Further studies of the cellular mechanism of **46b** showed that it arrested MCF-7 cells at the G2/M phase and induced apoptosis and necrosis^70^^,^^71^.

**Figure 24.**
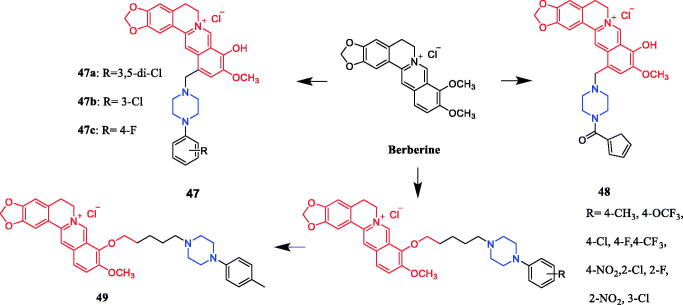
Chemical structures of berberine and its derivatives.

In recent years, several berberine derivatives were synthesised by Mistry et al., exerting anticancer activity against various human cancer cell lines. Conversely, a series of derivatives were synthesised by introducing various piperazine ring systems into 12-position of berberine. Mistry and co-workers reported that the berberine-piperazine derivative **47a** (Figure 24) demonstrated potent anticancer effects against HeLa cells, with an IC_50_ of 7.327 ± 0.08 µM, and was more potent than the lead compound berberine (IC_50_ = 13.42 ± 0.23 µM). The analysis of the results suggested that the derivatives may enhance their ability to bind to the target of the drug mainly through hydrophobic interaction, conjugation and hydrogen bonding on the 9-hydroxyl group. On further preparation, analog **47b** (Figure 24), demonstrating a substitution at the 3-position of the phenyl ring of piperazine moiety, was found to be the most effective, with an IC_50_ value of 3.983 ± 0.07 µg/mL against HeLa cells, and was more potent than the lead compound berberine (IC_50_ = 4.499 ± 0.08 µg/mL). In another report, compound **47c** (Figure 24) with a 3,4-dichlorophenyl piperazine entity was found to be the most active among derivatives tested, with an IC_50_ of 5.782 ± 0.55 µg/mL and CC_50_ of 320.7 ± 1.04 µg/mL, and the highest TI of 55.47 against HeLa cell lines. The potency of this compound was double that of the parent berberine which showed a TI of 28.16. The results clearly showed that the piperazine-based berberine analogues had stronger anti-tumour activity on the Caski cell line than Hela. It appeared that introduction of electron-withdrawing substituents (e.g. chloro- or fluoro-substituent) on the piperazine-linked benzene ring enhanced anti-tumour activity^72–74^. In 2016, berberine derivative **48** (Figure 24) with a furoyl piperazine substituent exhibited the highest anticancer activity against the CaSki cell line, with an IC_50_ value of 6.11 µM, CC_50_ value of 344.5 µM, and selectivity index of 56.39. The potency of this compound was double that of the parent berberine which showed a TI of 29.15. Conversely, a series of derivatives were synthesised by introducing various piperazine ring systems into the 9-position of berberine. Concerning the activity regarding the functional group attached to the piperazine ring, the order falls in the way alkyl > chloro > fluoro > nitro^75^. Analog **49** (Figure 24) with a 4-methylpiperazine substituent showed excellent anticancer property, with a therapeutic index (TI) of 58.53 (HeLa) and 48.76 (CaSki), better than that of a parent compound berberine with 27.41 and 25.84 of TI, and IC_50_ values of 5.595 ± 0.02 µg/mL and 6.716 ± 0.05 µg/mL, respectively^76^.

**Figure 25.**
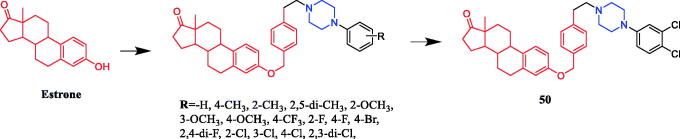
Chemical structures of oestrone and its derivatives.

Chen reported the synthesis of oestrone derivatives containing the piperazine moiety and evaluated their antitumor activities against several classical prostate cancer cell lines, including PC3, LNCaP, and DU145. Derivative **50** (R = 3,4-di-Cl) (Figure 25) showed significant cytotoxic actions against the LNCaP cell line (IC_50_ = 0.78 ± 0.34 µM), better than that of positive control finasteride (IC_50_ = 14.53 µM). and exhibited better α_1_-adrenergic receptor (AR) subtype selectivity over α_1B_ (α_1B_/α_1A_ ratio = 14.7). The SAR results showed that the ortho-substituted phenyl derivatives and the para-substituted phenyl derivatives were more effective on the cytotoxic activity of LNCaP and/or DU145 cells^77^.

**Figure 26.**
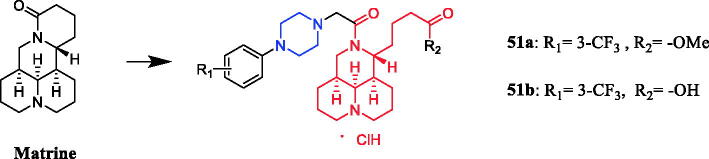
Chemical structures of matrine and its derivatives.

The in vivo antitumor assay revealed that matrine derivatives containing a piperazine moiety possessed improved therapeutic efficacy. Compound **51a** and **51b** (Figure 26) showed strong antitumor activity against human hepatoma Bel-7402 and colorectal carcinoma RKO cells (IC_50_ = 50.4 ± 1.4 µM and 48.3 ± 3.1 µM, 48.3 ± 2.5 µM and 51.3 ± 3.3 µM, respectively), proved to be of much better therapeutic efficacy than parent compound matrine (IC_50_ = 86.6 ± 2.6 µM and 84.3 ± 2.5 µM, respectively) and positive control cisplatin (IC_50_ = 92.5 ± 1.6 µM and 68.0 ± 1.3 µM, respectively). The SAR analysis indicated that the introduction of a piperazine moiety on matrine might significantly improve its antiproliferative activity, and the substituents and their position on phenyl of R1 and the variation of R2 could take a great role in antitumor activity^78^.

**Figure 27.**
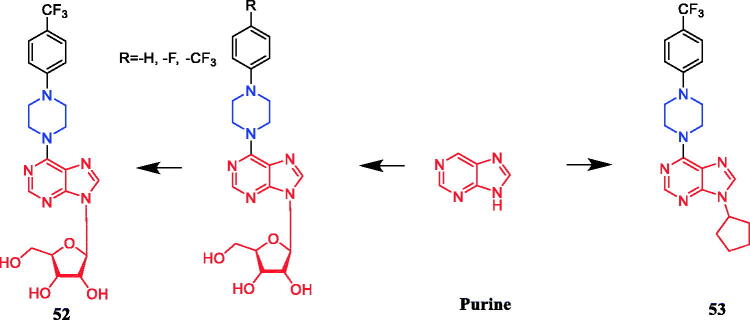
Chemical structures of purine and its derivatives.

Tuncbilek et al. developed purine ribonucleoside analogs and evaluated their antitumor activity. Compound **52** (Figure 27), containing 4-substituted piperazine in the substituent at N^6^, reportedly exhibited good antitumor activity against six different human tumour cell lines, with IC_50_ values between 5.2 and 9.2 µM. Further experiments also found that compound **52** may interfere with cellular ATP reserves by affecting cell kinase activity^79^. A series of purine derivatives were synthesised and evaluated against liver Huh7, breast T47D, and colon HCT116 carcinoma cells. Compound **53** (Figure 27) containing a piperazine moiety had better cytotoxic activities (IC_50_ ≤ 1 µM) than the nucleobase 5-FU and nucleosides fludarabine, cladribine, and pentostatine^80^.

**Figure 28.**
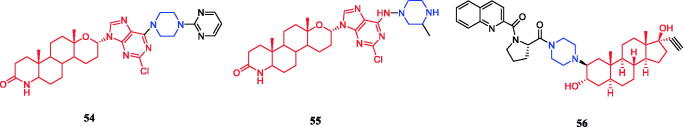
Chemical structures of steroid derivatives.

Huang et al. synthesised novel C6-piperazine substituted purine steroid-nucleosides analogs. Among them, compounds **54 and 55** (Figure 28) showed remarkable cytotoxicity against the PC-3 cell line (IC_50_ = 1.84 µM and 2.90 µM, respectively), better than that of parent compound purine nucleosides analogues with 11.19 µM of IC_50_. SAR suggesting that the substituents at C2- and C6-position of the purine ring and N4-position of piperazine moiety had a significant influence on the cytotoxicity^81^^,^^82^. Among the amino steroid derivatives synthesised by Ayan et al., compound **56** (Figure 28) with a piperazine-proline side-chain displayed potent antiproliferative activity among the five cancer cell lines evaluated (IC_50_ = 0.1, 0.1, 0.1, 2.0, and 1.1 µM for HL-60, MCF-7, T-47D, LNCaP, and WEHI-3, respectively), which was equivalent to the positive control doxorubicin (IC_50_ = 0.2, > 10, 0.7, 0.1, and 0.02 µM). Moreover, compound **56** showed extremely low cytotoxicity towards normal cells. In addition, compound 56 was injected subcutaneously in rats, plasmatic concentration was 151 ng/mL and 15.7 ng/mL, respectively at the 3 h and 12 h^83^.

**Figure 29.**
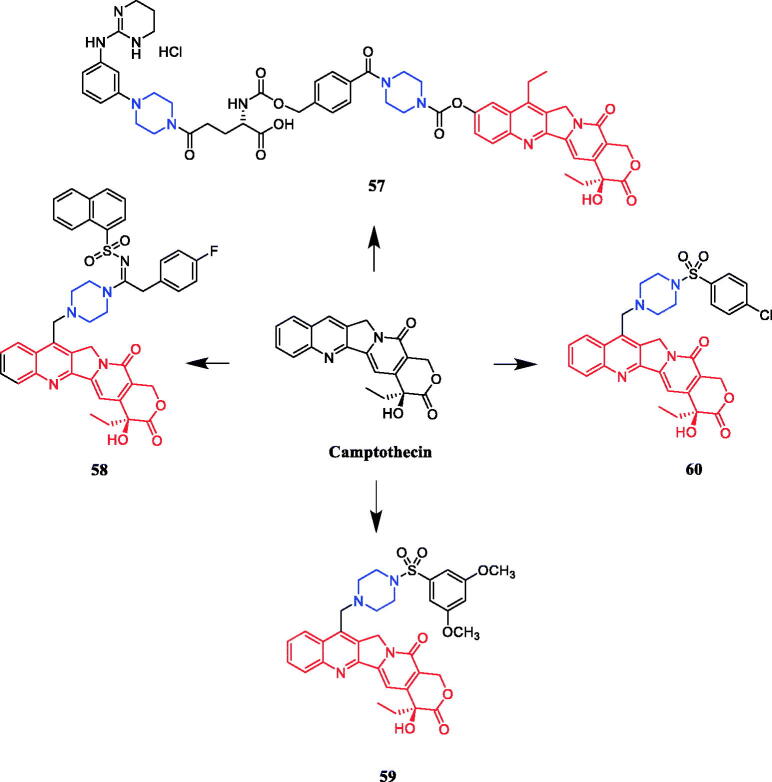
Chemical structures of camptothecin and its derivatives.

Piperazine carbamate was used to link camptothecin derivatives to prevent rapid hydrolysis. Compound **57** (Figure 29) demonstrated a potent affinity to integrin receptors and a potent ability to inhibit A2780 cells, with an IC_50_ value of 0.033 µM. Importantly, a significant increase in t_1/2_ for **57** vs irinotecan in rat plasma (13 h vs 45 min, respectively)^84^. Yang et al. reported the synthesis and cytotoxic activity of camptothecin derivatives incorporating piperazinyl-sulfonylamidine moieties. All derivatives were evaluated for their activity against five tumour cell lines (A-549, MDA-MB-231, MCF-7, KB and KB-VIN). SAR indicated that an aromatic group in R1 substituents is much less favourable than a short aliphatic group. The conjugation of the aromatic (R1) substituents with the sulphonyl moiety might disturb the electronic properties of ring B to a greater extent than alkyl groups. Moreover, the potency of the compounds with the aromatic substituents (R1) depended significantly upon the nature of the R2 substituent on the aromatic ring. Compound **58** (Figure 29) displayed the highest cytotoxicity against the MDR KB-VIN cell line (IC_50_ = 0.38 µM), while irinotecan lost activity completely against KB-VIN (IC_50_ > 20 µM)^85^. A series of 7-(N-[(substituted-sulphonyl) piperazinyl]-methyl)-camptothecin derivatives were designed and synthesised by Zhu and co-workers, assessed for their *in vitro* cytotoxicity against A-549, MDA-MB-231, KB, KB-VIN, and MCF-7 cell lines. Remarkably, compound **59** ((Figure 29, IC_50_ = 1.2 nM) and **60** ((Figure 29, IC_50_ = 20.2 nM) exhibited the highest level of cytotoxicity against the multidrug-resistant (MDR) KB-VIN cell line, which was equivalent to camptothecin (IC_50_ = 15.7 nM). SAR study suggested that both aromatic and aliphatic substituents on the sulphonyl-piperazinyl side chain at C-7 can promote potency to some extent, while selected variations of these substituents can greatly affect the activity. This study revealed that the integration of the sulfonylpiperazine modify into the 7-position of camptothecin is a potent strategy for discovering effective cytotoxic camptothecin derivatives^86^.

**Figure 30.**
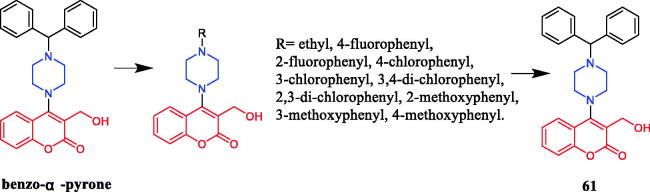
Chemical structures of benzo-α-pyrone and its derivatives.

New analogs of benzo-α-pyrone containing piperazine derivatives have been synthesised. Compound **61** (Figure 30) displayed the greatest activity against the WM266-4 (BRAFV600E) cell line, with an IC_50_ value of 1.35 µM. Unfortunately, the activity of **61** was not as good as that of the positive control vemurafenib (IC_50_ = 0.07 µM). Following the biological evaluation assays indicated that **61** could result in cellular apoptosis, marked DNA fragmentation, and G0/G1 phase arrest in melanoma cells. Moreover, docking simulation showed that compound **61** could bind well with the crystal structure of BRAFV600E at the active site. Compared with the compound in which piperazine is connected to the alkyl substituent, the aromatic-substituted compound inhibited BRAFV600 more strongly. Studies pointed out that the change in the position of the substituents on the piperazine-linked benzene ring may have little effect on the inhibitory activity, besides, the compounds substituted by the electron-donating groups on the benzene ring showed better inhibitory activity than the electron-withdrawing groups^87^.

**Figure 31.**
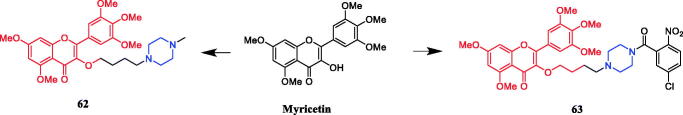
Chemical structures of myricetin and its derivatives.

Myricetin piperazine derivative **62** (Figure 31) exerted a moderate inhibitory effect against MDA-MB-231 cells (human breast adenoma cell) (IC_50_ = 3.87 ± 0.21 µM), was slightly lower than the positive control doxorubicin (IC_50_ = 2.01 ± 0.17 µM). The SAR indicated that all examined myricetin derivatives showed good activity against human breast cancer cell lines (MDA-MB-231 and Bcap-37) but poor activity against human gastric cancer cells (SGC-7901 and MGC-803 cell lines)^88^. Ruan et al. reported that myricetin derivative **63** (Figure 31) containing a piperazine moiety showed potent inhibitory activity against a human breast cancer cell line (MDA-MB-231) (Inhibition rate= 86.7% in 10 µmol/L), which was close to epirubicin (94.6%), In addition, through the analysis of the morphological effect on the target tumour, it can be seen that although the cytotoxicity of myricetin is small, its inhibitory effect on the tumour cells is not obvious; and the myricetin derivative after the introduction of piperazine amide has low cytotoxicity and can effectively inhibit the proliferation of tumour cells, which has good anti-tumour research value^89^.

**Figure 32.**
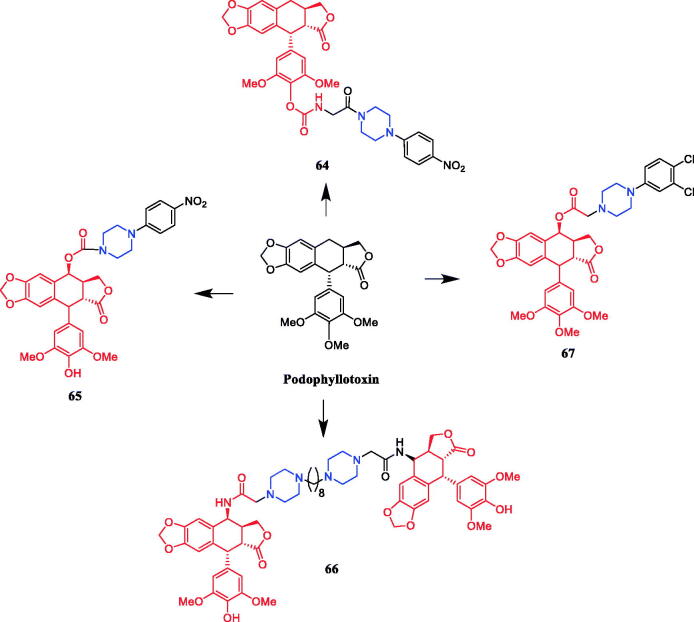
Chemical structures of podophyllotoxin and its derivatives.

Reportedly, derivative **64** (Figure 32) (1-*p*-nitrophenylpiperazinyl substituted derivative of deoxypodophyllotoxin) demonstrated the most active cytotoxicity against A-549, HeLa, and SiHa cells (IC_50_ = 0.102, 0.180, and 0.0195 µM, respectively), better than that of parent compound deoxypodophyllotoxin (IC_50_ = 0.470, 36.0, and 10.2 µM, respectively). Moreover, flow cytometric analysis indicated that the compound could arrest the cell cycle at the G1 phase in A-549 cells^90^. In 2013, demethylepipodophyllotoxins carbamate derivative **65** (Figure 32) was reported as the most promising compound synthesised in their study, resulting in cell cycle arrest in the G2/M phase and apoptosis in HeLa cells, presenting an IC_50_ value of 0.074 µM, better than that of positive control etoposide (IC_50_ = 2.91 µM). SAR indicated that the substituent of the amines markedly affected the activity profiles of this compound class, and the compounds with piperazines incorporated appear to be more potent than those with alkyl amines. Second, the compounds with amino acids incorporated appear to be less potent than those with p-nitrobenzylpiperzine directly and the compounds with L-amino acids incorporated appear to be more potent than those with D-amino acid. Lastly, compounds that contain only one substituent at the C-4 position showed superior activities in comparison with compounds simultaneously substituted at the C-4 and C-4′ position of 4′-demethylepipodophyllotoxin^91^. Five bis-epipodophyllotoxin compounds were designed using a structure-based design approach, showing strong evidence that these compounds targeted topoisomerase II in a variety of assays. Compound **66** (Figure 32) containing a linker with two piperazine rings exhibited the highest activity (IC_50_ = 0.016 µM), with an IC_50_ value 10-fold lower than etoposide (IC_50_ = 0.163 µM) against K562 cells. The IC_50_ values systematically decreased with an increase in methylene linker length and reached a minimum with compound **66** which contained eight methylenes. The IC_50_ values then coordinately increased when the linker length was increased to ten and twelve methylenes. These results suggested that an optimum chain length was in the order of eight methylene groups, and thus may be the optimum chain length needed for the bis-epipodophyllotoxins to achieve bisintercalation in the DNA within the topoisomerase IIbDNA ternary structure^92^. In 2017, Sun reported that podophyllotoxin piperazine derivative **67** (Figure 32) manifested prominent cytotoxicity against the MCF-7 cell line (IC_50_ = 2.78 ± 0.15 µM). Furthermore, compound **67** inhibited tubulin assembly without causing damage to non-cancerous cells, inducing apoptosis and cell cycle arrest at the G0/M phase in MCF-7 cells. It can be seen from the experimental data that almost all synthesised compounds showed anti-cancer activity, the substituents on the aromatic ring had a significant effect on the activity. According to the SAR, it was clearly found that compounds with substitution on the phenyl ring of the phenylpiperazine group in general exhibited inhibitory activities, electron-withdrawing groups (F, Cl, NO_2_, CF_3_) > electron-donating groups (OMe, Me), however, the modification of the 2 or 4 positions of the benzene ring resulted in a decrease in potency^93^.

**Figure 33.**
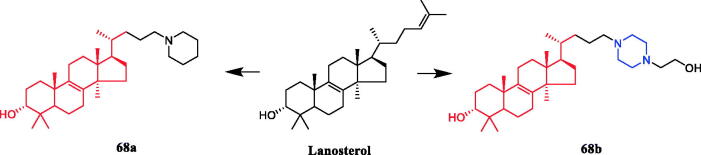
Chemical structures of lanosterol and its derivatives.

The synthesis of a range of novel 24-amino-25,26,27-trinorlanost-8-ene derivatives including 24-piperadino-trinorlanost-8-enes, 24-piperazino-trinorlanost-8-enes, 24-morpholino-trinorlanost-8- enes, and 24-diethylamino-trinorlanost-8-enes is reported and their cytotoxic and apoptotic potential evaluated in U937 cell lines. SAR indicated that morpholine, aniline, piperidine or diethylamine functionality was introduced at C-24 with acetate or hydroxyl functionality at C-3. The order of potency of these derivatives was piperidine > diethylamine > morpholine > aniline, with hydroxyl functionality at C-3 proving more potent than the acetylated counterpart. The piperidine derivative **68a** (Figure 33) was revealed as a potential anticancer agent against human leukemic monocyte lymphoma U937 cells, presenting an IC_50_ value of 1.9 µM, which was equivalent to the positive control etoposide (IC_50_ = 1.5 µM). Besides, **68b** (Figure 33) with piperazine moiety had an IC_50_ value of 2.7 µM^94^.

**Figure 34.**
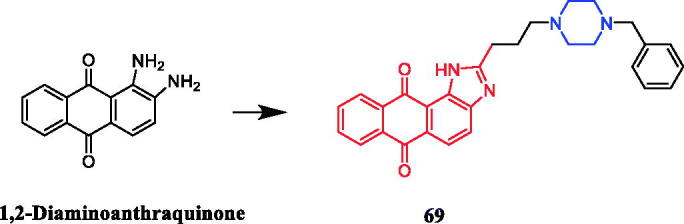
Chemical structures of anthraquinone and its derivatives.

Chen and co-workers synthesised a series of anthraquinone derivatives to determine their potential telomerase inhibitory activities. It was found that compound **69** (Figure 34) with benzylpiperazine was found to show the best selectivity towards repressing hTERT expression in H1299 cells among these tested compounds. And compound **69** showed moderate potency against PC-3 with IC_50_ = 16.0 µM. Furthermore, SAR analysis indicated that the piperazine linker might be a significant moiety for telomerase inhibition or hTERT repression^95^.

**Figure 35.**
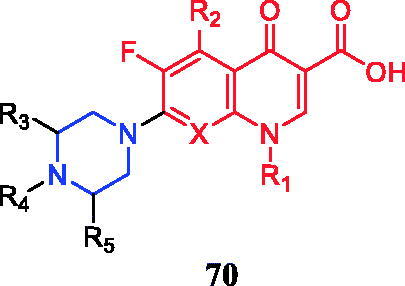
Chemical structures of piperazinylquinolone skeleton.

Piperazinylquinolone **70** (Figure 35) was considered a starting point for the design and development of new anticancer agents. Chemical modification of N-4 in the piperazine ring and the carboxylic acid at C-3 could present sufficient flexibility^96^.

**Figure 36.**
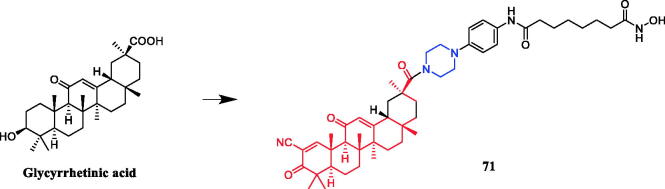
Chemical structures of glycyrrhetinic acid and its derivatives.

Pterins are a class of fused N-heterocyclic compounds containing pyrimidine and pyrazine rings, which widely existed in natural products and bioactive molecules^97^. In a study presented by Huang et al., a piperazine linker was identified as the best hybrid approach for glycyrrhetinic acid and hydroxamic acid. Conjugate **71** (Figure 36) displayed effective cytotoxicity against cancer cells inducing apoptosis, with IC_50_ values of 0.47 ± 0.09 µM and 0.37 ± 0.07 µM, respectively, against PC-3 and HL-60 cell lines. Notably, compound **71** showed better potency than SAHA (IC_50_ = 1.81 ± 0.15 µM and 0.42 ± 004 µM), while exhibiting superior inhibitory efficacy comparing to the parental compound CDODA-Me (IC_50_ = 1.68 ± 0.28 µM and 1.35 ± 0.16 µM) as well. The preliminary pharmacokinetic (PK) property of compound **71** was evaluated in male Sprague-Dawley (SD) rats. Compound **71** at 5 mg/kg was intravenously (iv) administered to rats (*n* = 3). Compound **71** demonstrated reasonable PK profiles with a high half-life (*T*_1/2_) of 16.75 h and maximum concentration (*C*_max_) of 10753.33 ng/mL. The results suggested that the piperazine substituted 5,8-dihydropteridine-6,7-dione frameworks may be regarded as new chemotypes for designing effective antitumor agents against gastric cancer cells^98^.

**Figure 37.**
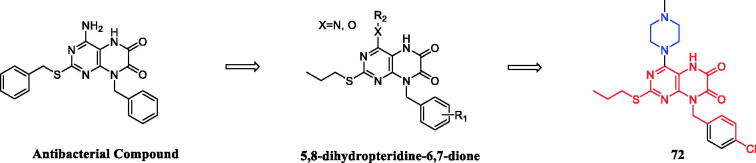
Chemical structures of 5,8-dihydropteridine-6,7-dione and its derivatives.

Geng et al. reported the synthesis and preliminary biological evaluation of 5,8- dihydropteridine-6,7-diones. The SARs studies highlighted the importance of the piperazine substituted 5,8-dihydropteridine-6,7-dione frameworks for the activity and revealed essential structural elements. Among these derivatives, compound **72** (Figure 37) displayed the most potent and broad-spectrum proliferative inhibition against evaluated cell lines (HeLa, CaSki, MGC-803, and SKOV-3 cancer cell lines) and was sensitive to the MGC-803 cell line, with an IC_50_ value of 8.78 µM, slightly more potent than 5-fluorouracil (IC_50_ = 14.15 µM). Preliminary mechanistic studies showed that compound **72** could inhibit the colony formation and migration of MGC-803 cells. Besides, flow cytometry analysis showed that compound **72** concentration-dependently induced apoptosis of MGC-803 cells^99^.

A series of disubstituted C20’-urea derivatives of vinblastine were prepared by Barker et al. and were examined across a panel of 15 human tumour cell lines. N-methylpiperazine was introduced to increase the polarity of designed compounds^100^.

**Figure 38.**
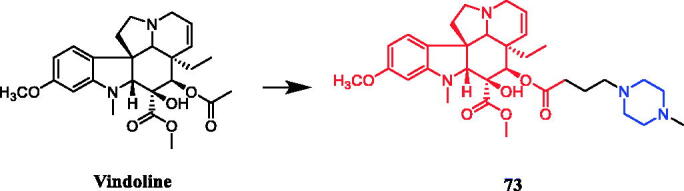
Chemical structures of vindoline and its derivative.

Vindoline conjugated with N-methylpiperazine (compound **73**, Figure 38) demonstrated a significant antiproliferative effect, with IC_50_ values of 9.36 µM, 14.10 µM and 25.49 µM, respectively, against HeLa, MCF-7 and MDA-MB-231 cell lines, although the monomer vindoline was inactive (IC_50_ > 30 µM). In addition, the experimental results suggested that the compound of ventolin and amino acid, tryptophan methyl piperazine and N-methyl piperazine have significant anti-proliferative effects. Concerning structure–activity relationships, it seems that the 1,2,3‐triazole and morpholine moieties could not improve the efficacy of vindoline at all^101^.

**Figure 39.**
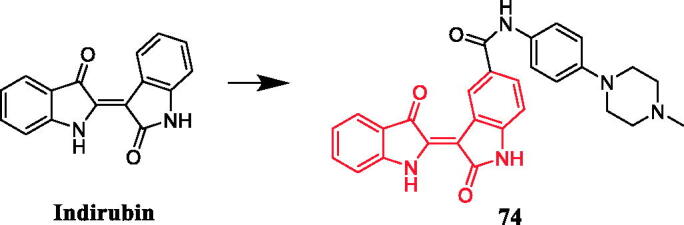
Chemical structures of indirubin and its derivative.

A series of indirubin derivatives were designed to improve water solubility and antitumor activity. Among them, compounds containing a piperazine moiety exhibited significant inhibitory activity against human large cell lung cancer cells (LXFL529L cells). Compound **74** (Figure 39) carrying a methylpiperazino-phenylamino substituent attached to 5-carboxyindirubin, with a water solubility of 520 mg/L, displayed the most potent antitumor activities, with an IC_50_ value of 0.54 µM, better than that of Positive control 5-carboxyindirubin (IC_50_ = 7 µM). When the N-methylpiperazinyl substituent on the benzene ring was changed to dimethylamino, although the water solubility increased, the activity of inhibiting cell proliferation decreased. In addition, the phenyl moiety of **74** replaced by an aliphatic chain also produced a similar effect^102^.

**Figure 40.**
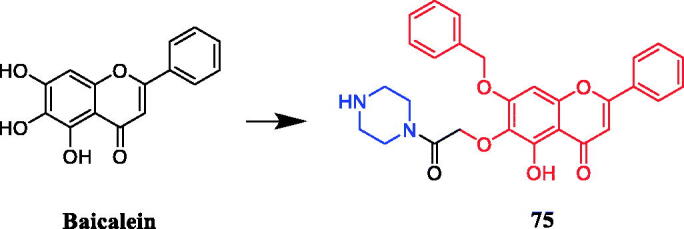
Chemical structures of baicalein and its derivative.

Several baicalein-piperazine derivatives showed moderate anti-angiogenic activity, with weak toxicity on normal tissues. Notably, compound **75** (Figure 40), with the piperazine acetamide group at 6-position, was the most active compound on human umbilical vein endothelial cell (HUVEC) proliferation, migration, and tube formation *in vitro*. Moreover, consistent with the anti-angiogenic activity, compound **75** significantly inhibited the growth of A549 cells (IC_50_= 4.73 µM). The data indicated the introduction of an alkyl alcohol substituent at the 6 positions could increase the anti-angiogenic activity, and shortening the chain length reduced toxicity. In addition, regarding the 6-O-acetamide substituted scaffold, the substitution of a benzyl group for the phenolic hydroxyl group did not significantly improve the effect on inhibiting angiogenesis, while the compound **75** with a piperazine group showed the strongest anti-angiogenic activity^103^.

**Figure 41.**
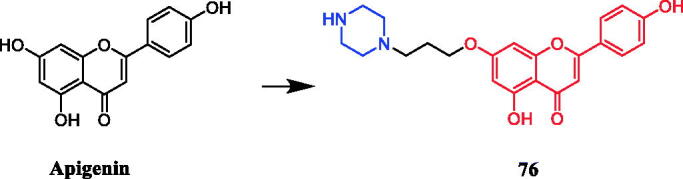
Chemical structures of apigenin and its derivative.

Liu et al. reported that apigenin-piperazine derivative **76** (Figure 41) possessed active antiproliferative activity with the lowest IC_50_ values of 16 µg/mL, 11 µg/mL, 25 µg/mL and 32 µg/mL against A549, HeLa, HepG2, and MCF-7 cancer cells, more potent than parent compound apigenin (IC_50_ = 1740 µg/mL, 450 µg/mL, 460 µg/mL and > 2000 µg/mL). SAR analysis indicated that compared with the compounds with 2-carbon spacer, 3-carbon-spacer-containing compounds, showed a slight increase in antiproliferative activity against the four cancer cell lines. This result may have been caused by the elongation of the alkyl side chain from two to three C-atoms that retained the modulating activity, with C3-bridge derivatives being the most active. The compounds containing N-heterocyclic-ring amino side chains displayed better activities against the four cancer cell lines than those containing alkyl amino side chains. This result may be attributed to the increasing lipophilicity of these compounds, which could influence their ability to reach the target via transmembrane diffusion^104^.

**Figure 42.**
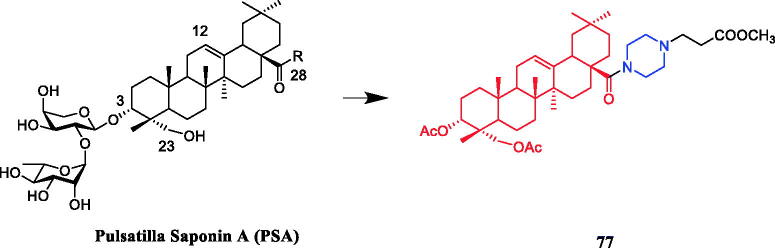
Chemical structures of pulsatilla saponin A and its derivatives.

Twenty Pulsatilla saponin A (PSA) derivatives with C ring, C-28, or C-3 modifications were synthesised and evaluated for cytotoxic activities against A549, MCF-7, MDA-MB-231, KB, SMMC-7721, BGC-823, and KB-VIN cell lines. Compound **77** (Figure 42) with the piperazine ring at the 28-COOH position exhibited better cytotoxic activity (IC_50_= 4.68 µM) and lower haemolytic toxicity (HD_50_ *>* 500 µM) against the A549 cell line when compared with PSA (IC_50_= 22.87 µM, HD_50_ =3.86 µM). The data indicated that the carbon ring (especially the C-12/C-13 double bond) might play an important role in the cytotoxicity and haemolytic toxicity of prostate-specific antigen. The 28-COOH may cause haemolysis of red blood cells. In addition, the fully acetylated sugar chain at position 1 showed weaker cytotoxicity than PSA, indicating that the sugar hydroxyl group is important for the cytotoxicity of PSA. Further molecular biology studies showed that compound **77** might cause G1 cell cycle arrest^105^.

### 2.2. Antibacterial activity

**Figure 43.**
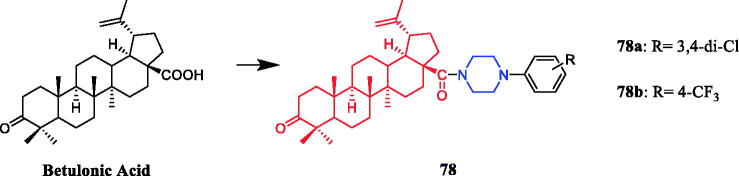
Chemical structures of betulonic acid and its derivatives.

Piperazine derivatives developed by Chue et al. were assessed for their antibacterial potential, demonstrating the synthesis of betulonic acid amides with N-derivatives of piperazine and their antibacterial activities. Compounds **78a and 78b** (Figure 43) inhibited bacterial growth in all tested cultures. The diameter of the growth inhibition zone was in the range of 13 − 16 mm for five of the studied test cultures, which is better than the positive control gentamicin (16 − 21 mm)^106^.

**Figure 44.**
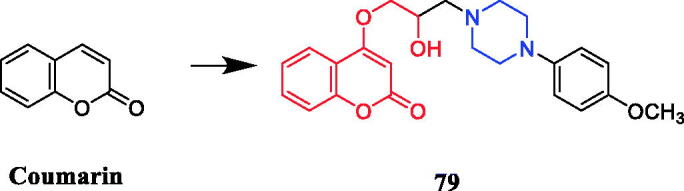
Chemical structures of coumarin and its derivatives.

Wang synthesised coumarin derivatives containing a piperazine skeleton as potential antibacterial agents. Compound **79** (Figure 44) demonstrated the most potent antibacterial activity [minimum inhibitory concentration (MIC) = 0.236 µg/mL for *Bacillus subtilis*; MIC = 0.355 µg/mL for *Staphylococcus aureus*] compared with the positive control Penicillin G (MIC = 0.752 µg/mL and 1.685 µg/mL). What’s more, it showed the most potent activity against SaFabI with IC_50_ of 0.57 µM. The SAR revealed the activity of compounds with substituents on the benzene ring is better than that of unsubstituted compounds, moreover, the para-substituted compounds showed stronger antibacterial activity than the meta- and ortho-substituted compounds^107^.

**Figure 45.**
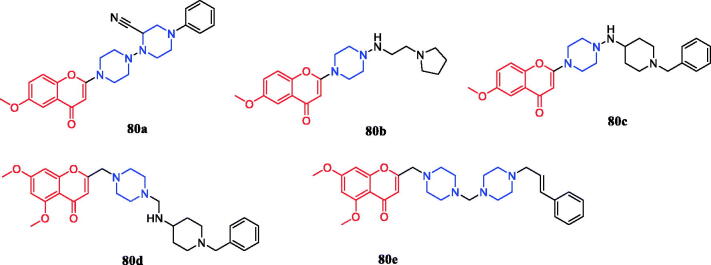
Chemical structures of flavone derivatives.

Flavone derivatives **80a**–**80e** (Figure 45) with piperazine rings were found to demonstrate more potent antibacterial activity, showing even 2 to 2.5-fold more potency than that of standard ciprofloxacin and miconazole at the same MIC value of 10 µg/mL. It was observed that the presence of amino alkyl, cyano or alkenyl alkyl group on piperazine ring seemed to be favourable for the high antimicrobial activity, however, the mere presence of electron donor group on the piperazine ring had very little or nothing to do with antimicrobial activity^108^.

**Figure 46.**
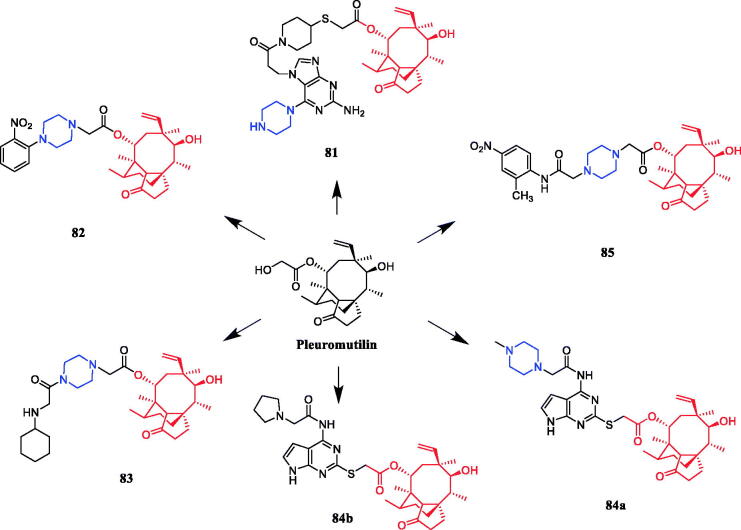
Chemical structures of pleuromutilin and its derivatives.

In recent years, several pleuromutilin derivatives containing piperazine moieties were designed and synthesised. Pleuromutilin analog **81** (Figure 46) was designed and synthesised with a purine ring as a polar and water solubilising group. In further investigation, analog **81** with piperazine displayed superior *in vitro* and *in vivo* antibacterial activity against some Gram-positive strains, including drug-resistant pathogens. Analog **81** with good solubility in water showed remarkable antibacterial activity against all Gram-positive pathogens, including methicillin-resistant *S. aureus* (MRSA), penicillin-resistant *Streptococcus pneumoniae* (PRSP), and vancomycin-resistant *enterococci* (VRE), except for *Enterococcus faecalis*. Importantly, analog **81** exhibited the most potent antibacterial activity against susceptible and resistant *S. pneumoniae*, with a MIC of 0.063 µg/mL, appeared as a possible alternative to the weak marketed antibacterial agents macrolide erythromycin (MIC > 128 µg/mL), tetracycline minocycline (MIC = 8 µg/mL), and quinolone levofloxacin (MIC = 8 µg/mL). Interestingly, **81** and glycopeptide vancomycin (VCM) exhibited superior ED_50_ values (about 1–2 mg/kg) when given intravenously regardless of the frequency of dosing (once or twice), indicating the potential of **81** as an antibacterial agent with efficacy equal to that of VCM^109^. A series of novel pleuromutilin derivatives having a piperazine ring were synthesised under mild conditions. In the ortho-, meta- and para-position of the benzene ring, electron withdrawing and donating groups were introduced to explore SAR. The ortho-NO_2_ substituted derivative **82** (Figure 46) was found to be the most active compound against S. aureus (MIC = 0.0625 µg/mL) among a series of pleuromutilin derivatives, better than the positive control tiamulin (MIC = 0. 25 µg/mL). The results revealed ortho-substituted benzene derivatives have the greatest impact on activity compared with para and meta substitutions, for example, replacement of the nitro group in compound **82** with chlorine group at the same position, which resulted in dramatic decrease in antibacterial activity. The meta-substituted benzene derivatives with CH_3_O–, CH_3_–, and NO_2_– substituents showed enhanced antibacterial activities, all the para-substituted benzene derivatives bearing methoxyl, methyl, hydroxyl, nitro, and chlorine substituents exhibited moderate antibacterial activities. The compound-bearing nitro group in the para-position showed higher activity compared to other para-position substituted derivatives. The para-position appeared to have less influence on the antibacterial activity of these pleuromutilin derivatives bearing a phenyl piperazine^110^. In 2015, derivative **83** (Figure 46) was revealed remarkable activity against *S. aureus* (ATCC25923; MIC of 0.125 µg/mL), which is equal to the control compound tiamulin. The antibacterial activities of **83** to *Streptococcus suis* (MIC of 2 µg/mL), *Streptococcus agalactiae* (MIC of 0.5 µg/mL), and *Streptococcus dysgalactiae* (MIC of 0.5 µg/mL) were also excellent compared with the control drug erythromycin (MIC of > 128 µg/mL). Further, the results preliminary confirmed that the activity of secondary amine is better than that of a tertiary amine. A molecular docking study indicated that **83** could bind to the active pocket of the ribosome with seven hydrogen bonds^111^. In 2018, pleuromutilin-piperazine derivative **84a** (Figure 46) exhibited moderate antibacterial activity against methicillin-sensitive *S. aureus* (MSSA) with MIC of 0.125 µg/mL, that even more active than the comparator valnemulin (MIC = 0.25 µg/mL). However, the activity against *P. multocida* was also examined, and the most efficient compound **84b** proffered identical MIC (16 µg/mL) as valnemulin. In addition, the results provided by the dose-response study demonstrated **84b** could supply infected mice with a 70% survival rate at a dose of 40 mg/kg via intragastric (i.g.) administration^112^. Furthermore, in 2020, pleuromutilin derivative **85** (Figure 46) was revealed as the most potent derivative against *S. aureus* and *Staphylococcus epidermidis*, with a MIC value of 0.0625 µg/mL, showed an almost two-fold increase in activity in comparison with that of reference drugs, and an eight-fold increase in activity against *Enterococcus faecalis* compared to that of tiamulin. Furthermore, the docking study revealed that derivative **85** could interact with the ribosome pocket with more bonds when compared with tiamulin^113^.

**Figure 47.**
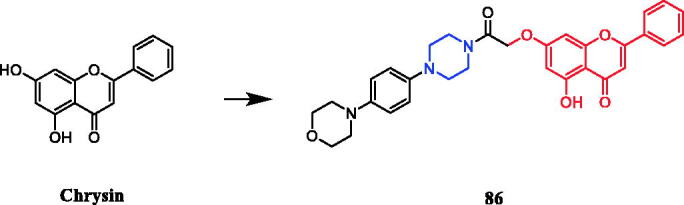
Chemical structures of chrysin and its derivatives.

Reportedly, a new series of piperazinyl-substituted chrysin derivatives have been designed and synthesised. Among the synthesised compounds, analogs accommodating morpholine, piperidine, and electron-donating groups at the benzene ring showed better antibacterial activities than those that possess electron-withdrawing substituents. The chrysin containing morpholine compound **86** (Figure 47) displayed the most potent inhibitory activity against Escherichia coli FabH (IC_50_ = 5.78 ± 0.24 µM), comparing to the control positive drugs penicillin G (7.56 ± 0.30 µM). In the antibacterial activity assay, **86** was found to be the most active against *S. aureus* and *E. coli* (MIC = 1.25 ± 0.01 and 1.15 ± 0.12 µg/mL, respectively), compared with the control positive drugs (MIC = 1.39 ± 0.02 and 1.82 ± 0.47 µg/mL, respectively)^114^.

**Figure 48.**
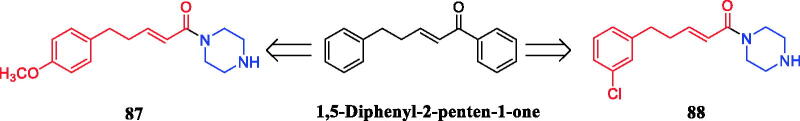
Chemical structures of 1,5-diphenyl-2-penten-1-one and its analogs.

A series of novel 1,5-diphenyl-2-penten-1-one analogs with a piperazine moiety were synthesised and evaluated for their antifungal and larvicidal activities. Among them, the most potent compound **87** (Figure 48) demonstrated 100% mortality against mosquito larvae at a concentration of 1 mg/L. Compound **88** (Figure 48), with a 3-position chlorine substituted phenyl group, exhibited the highest activity with an EC_50_ value of 0.075 mM against *Pythium aphanidermatum*, superior to lead compound 1,5-Diphenyl-2-penten-1-one (0.39 mM). Initial SAR analysis showed that a methyl group had significant effects on the biological activities of these compounds, the compounds with N’-unsubstituted piperazine exhibited much better antifungal activities and larvicidal activity against mosquitoes than the compounds with N’-methylated piperazine. In addition, the larvicidal activity against mosquitoes had a sharp decline when the substituent on the benzene ring was changed from 4-position to 2 or 3-position^115^.

**Figure 49.**
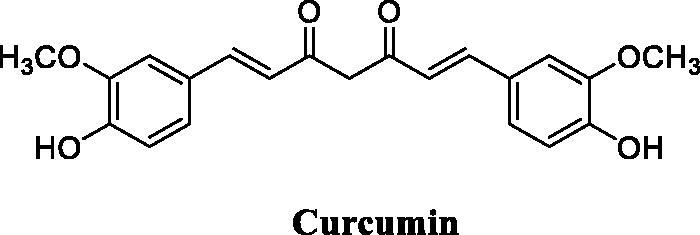
Chemical structures of curcumin.

Eight novel curcumin derivatives were synthesised using the Mannich reaction and evaluated for their antibacterial activities; two derivatives containing a piperazine ring showed remarkable mildew resistance (Figure 49)^116^.

**Figure 50.**
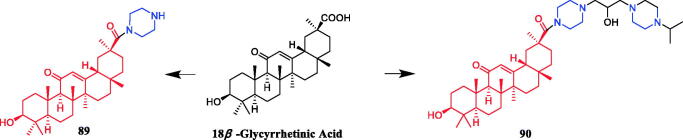
Chemical structures of glycyrrhetinic acid and its derivatives.

In 2011, glycyrrhetinic acid derivative **89** (Figure 50) containing a piperazine ring displayed excellent anti-mycobacterial properties against the drug-susceptible and drug-resistant *Mycobacterium bovis*. More importantly, it exhibited synergistic effects with the first‐line drugs isoniazid (INH), rifampicin (RIF) and streptomycin (SM) against clinical *M. bovis* isolates, including drug‐resistant strains. In the presence of **89**, MICs for the first‐line agents resulted in a 4–16‐fold decrease for INH ((fractional inhibitory concentration index (FICI) 0.094–0.266), RIF (FICI 0.114–0.313) and SM (FICI 0.094–0.281). Additionally, the MICs of PGA alone showed significant decreases ranging from 8‐ to 16‐, 8‐ to 64‐ and 8‐ to 128‐fold in the presence of INH, RIF and SM, respectively. These findings indicate that **89** might serve as potential therapeutic compounds for future antimycobacterial drug development^117^. Xiang et al. synthesised a series of novel 18*β*-glycyrrhetinic piperazine amides to evaluate *in vitro* and *in vivo* antibacterial activities against phytopathogens *Xanthomonas oryzae* pv. oryzae (Xoo) and *X. axonopodis* pv. citri (Xac), as well as induced apoptotic behaviours. Screening results revealed that all designed compounds were bioactive against Xoo and Xac. Among them, compound **90** (Figure 50) bearing an isopropyl tail presented the most potential antibacterial efficiency, with EC_50_ values of 2.28 and 1.42 µg/mL against Xoo and Xac, respectively, which is far better than lead compound 18β-glycyrrhetinic acid (EC_50_ > 400 µg/mL), positive control bismerthiazol (EC_50_ = 92.6 µg/mL and NT) and thiodiazole copper (EC_50_ = 121.8 µg/mL and 77.0 µg/mL). Pharmacophore studies suggested that the synergistic effect of glycyrrhetinic acid skeleton and N-containing scaffolds was essential for the antibacterial activity. Further studies revealed that compound **90** could induce the generation of excessive ROS in tested pathogens^118^.

**Figure 51.**
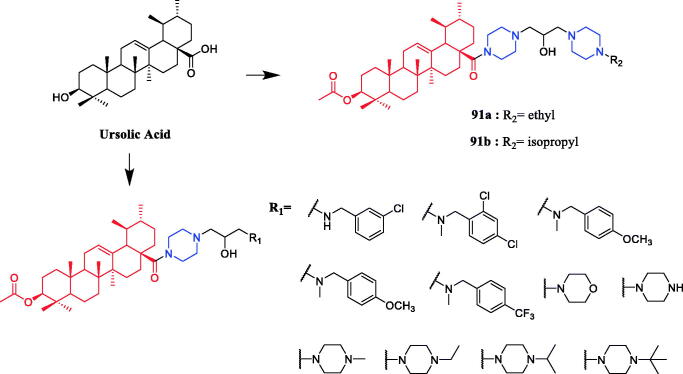
Chemical structures of ursolic acid and its derivatives.

In another study, piperazine-tailored ursolic acid hybrids **91a** (Figure 51) exhibited remarkable antimicrobial activity against Xoo and Xac (EC_50_ values were 0.37 and 1.08 µg/mL, respectively), which is far better than lead compound ursolic acid (EC_50_ > 400 µg/mL), positive control bismerthiazol (EC_50_ = 92.6 µg/mL and NT) and thiodiazole copper (EC_50_ = 121.8 µg/mL and 77.0 µg/mL). Studies have shown that the hydroxyl group in the derivative structure had a positive role in promoting antibacterial action, on the contrary, the elimination of the N-ethyl piperazinyl or N-isopropyl piperazinyl group dramatically reduced antibacterial activity. Importantly, compounds **91a a**nd **91b** (Figure 51) could manage bacterial blight *in vivo* in pot experiments. A preliminary finding revealed that the synthesised compounds could disrupt the equilibrium of the redox system in tested pathogens, leading to excessive ROS accumulation, which induced the apoptosis-like effect in tested pathogens^119^.

**Figure 52.**
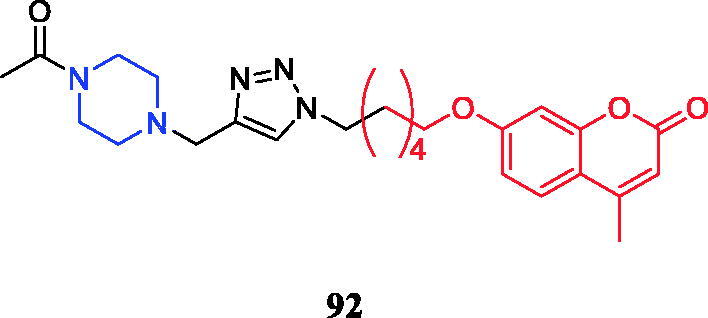
Chemical structures of chromen derivatives.

Amongst a series of novel 2H-chromen-2-one derivatives bearing 1,2,3-triazole moiety, compound **92** (Figure 52) containing N-acetylpiperazine moiety was found to be most effective against *Pseudomonas aeruginosa* (zone of inhibition in 17.1 mm), which is equivalent to the standard (zone of inhibition in 18 mm). Based on, *in silico* pharmacokinetic studies, compounds **92** was identified as lead compounds for future investigation due to their lower toxicity, high drug score values and good oral bioavailability as per OECD guidelines^120^.

### 2.3. Anti-inflammatory activity

Inflammation is a non-specific immune response to infection, tissue stress, or some other form of injury^121^. Almost all modern human diseases are related to chronic inflammation^122^. Moreover, glucocorticoids and non-steroidal anti-inflammatory drugs currently in clinical use have serious gastrointestinal side effects^123^^,^^124^. Therefore, researchers have developed a series of anti-inflammatory piperazine derivatives based on natural products.

**Figure 53.**
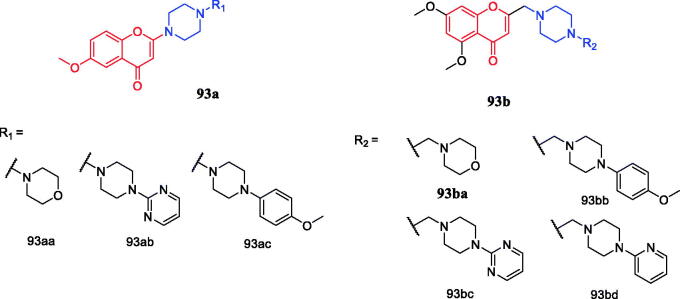
Chemical structures of flavone derivatives.

A series of flavone piperazine derivatives were synthesised and evaluated for their *in vitro* anti-inflammatory activity. Among all the compounds screened, compounds **93a**–**93b** (Figure 53) showed promising TNF-α and IL-6 inhibitory activity. Furthermore, Compound **93ba** (Figure 53) exhibited excellent inhibition against tumour necrosis factor-α (TNF-α) and interleukin (IL)-6 (up to 65–87% TNF-a and 70–93% IL-6 inhibitory activity) at a concentration of 10 µM with reference to standard dexamethasone (71% TNF-a and 84% IL-6 inhibitory activities at 1 µM. Notably, the presence of highly electron-rich groups such as methoxy, pyrimidyl, morpholine on piperazine as well as homologation of chromone and piperazine moiety had strong relevance to the anti-inflammatory activity^108^.

**Figure 54.**
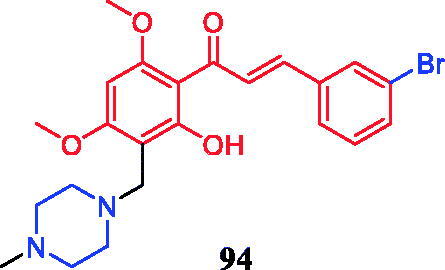
Chemical structures of chalcones derivative.

Compound **94** (Figure 54) demonstrated significant activity against β-glucuronidase (inhibition rate = 75.43% at 1 mM concentration), which was better than that of natural chalcone (NC, (E)-1–(2-hydroxy-4,6-dimethoxyphenyl)-3-phenylprop-2-eb-1-one, inhibition rate = 28.42% at 1 mM concentration). Further results indicating that chalcones with N-methyl piperazine methyl and piperidine methyl substitutions appear to be crucial for *β*-glucuronidase inhibition, and N-methyl piperazine group substitution at 3 positions of terminal benzene ring was found to be more suitable for the reduction of DPPH. Compound **94** was revealed the highest antioxidant activity in the DPPH free radical scavenging assay at 1 mM concentration (DPPH reduction rate = 37%) in the synthesised compound, far exceeding NC (DPPH reduction rate = 16.67%) but still weaker than quercetin (DPPH reduction rate = 86.30%)^125^.

**Figure 55.**
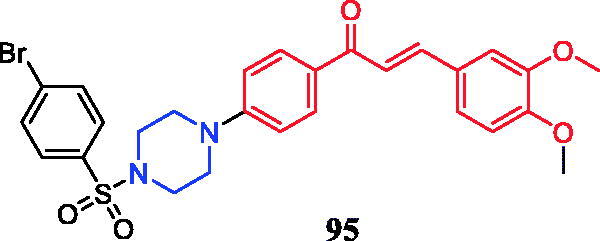
Chemical structures of chalcone-biscoumarin derivative.

Novel chalcone-biscoumarin hybrids were synthesised by exploiting the anti-inflammatory potential of natural chalcone derivatives. All hybrid molecules were evaluated for in vivo anti- inflammatory activity using the in vivo para-xylene-induced mice ear-swelling model. Particularly, hybrid **95** (Figure 55) showed the most active anti-inflammatory activity (inhibition rate, 75.46%), which exceeded that of aspirin (inhibition rate, 64.91%) and was equal to that of celecoxib (inhibition rate, 77.03%). Preliminary SAR analysis indicated that substitution of aryl-piperazine or aryl-sulphonyl-piperazine with an electron-withdrawing group demonstrated an advantage over the electron-donating group, improving the anti-inflammatory activity^126^.

**Figure 56.**
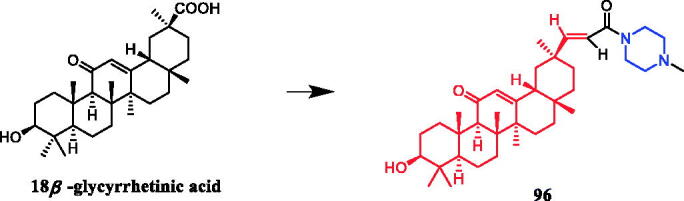
Chemical structures of glycyrrhetic acid and its derivative.

In a study performed by Li et al., compound **96** (Figure 56) with a piperazine unit exhibited the most potent nitric oxide (NO) and IL-6 inhibitory activity (IC_50_ = 13.3 µM) in the tested unsaturated glycyrrhetic acid derivatives. Moreover, compound **96** significantly reduced lipopolysaccharide (LPS)-induced inducible nitric oxide synthase (iNOS) and cyclooxygenase (COX)-2 expression, as well as IL-6 production, through the MAPK and NF-κB signalling pathways^127^.

### 2.4. Antioxidant activity

**Figure 57.**
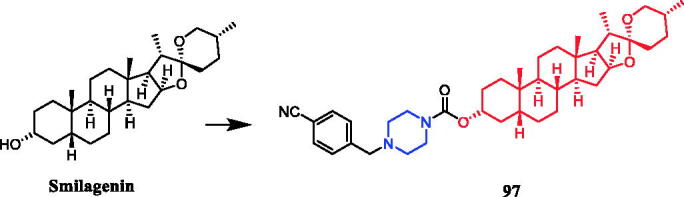
Chemical structures of smilagenin and its derivatives.

In 2019, smilagenin derivative **97** (Figure 57) was evaluated for its potential neuroprotective effects, and revealed improved cell viability against H_2_O_2_-induced damage, inhibited NO production, and demonstrated strong antioxidant activity. The protective effect of compound **100** at a concentration of 10 µM (Cell viability = 40.5 ± 2.3%) against the oxidative stress triggered by H_2_O_2_ in SH-SY5Y cells was more effective than that of positive control N-acetyl-L-cysteine at 1 mM (Cell viability = 37.1 ± 2.5%). The activity results revealed benzyl-substituted piperazine formate derivatives showed the potent neuroprotective activity^128^.

β-elemene piperazine derivatives were synthesised and evaluated for their inhibitory abilities. The data results indicated that these derivatives induced apoptosis through ROS production and reduced c-FLIP levels, activating both death receptor-mediated and mitochondrial-mediated apoptotic pathways^129^.

**Figure 58.**
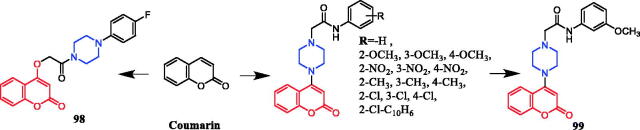
Chemical structures of coumarin and its derivatives.

Coumarin derivative **98** (Figure 58) exhibited significant activity (IC_50_ = 2.1 µM) and good selectivity for AChE with respect to butyrylcholinesterase. Structure-activity relationship studies have shown that the anti-acetylcholinesterase activity of the compound was affected by the type of cyclic amine on the 2-oxo- or 4-oxoalkoxycoumarinbackbone^130^. Coumarin piperazine derivative **99** (Figure 58) was found to be a potent AChE inhibitor, with an IC_50_ value of 4.9 µmol/L against human AChE enzyme (hAChE), while the IC_50_ values of reference drug ensaculin and doneprazil were 0.36 ± 0.01 µmol/L and 0.11 ± 0.01 µmol/L, respectively. Among the newly synthesised analogs, the most effective compound was **102**, having a methoxy group at the meta position. If the methyl group is attached in the ortho and para positions, the activity was further reduced. In addition, when the ortho-substituted methyl group of the compound was shifted to the para and meta positions, the inhibitory activity declined. Compounds showed better inhibitory activity when nitro was substituted at the meta position, or remarkably decreased if nitro shifted to the ortho and para positions. Subsequently, a SAR study using the molecular field method revealed that the inhibitory mechanism may be attributed to the hydrophobic field and positive charge centre conferred by the coumarin and piperazine moieties^131^.

**Figure 59.**
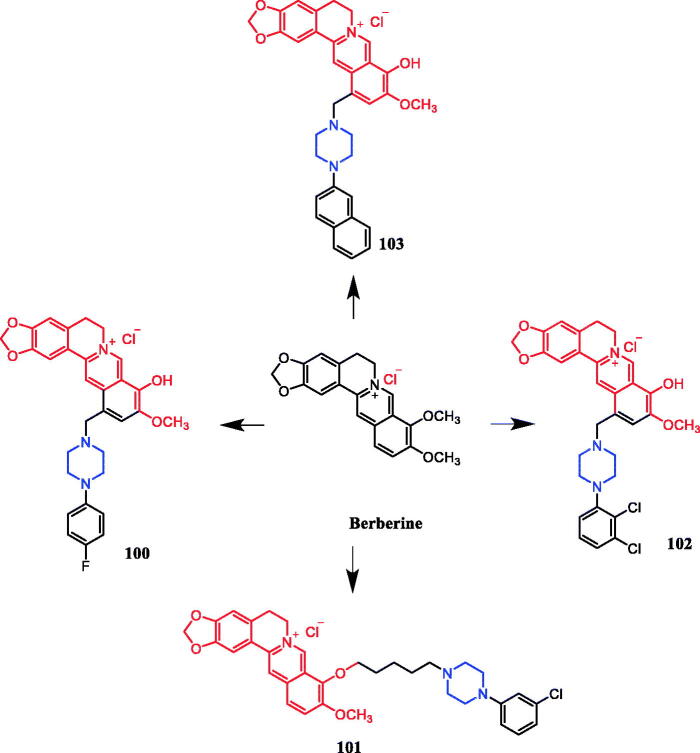
Chemical structures of berberine and its derivatives.

In the previous section, it was stated that a series of berberine derivatives were successively reported by Mistry et al, simultaneously presenting the anticancer and antioxidant activities. Compound **100** (Figure 59) showed remarkable antioxidant ability in the ABTS assay, with an IC_50_ of 4.644 µg/mL. In another report, analog **101** (Figure 59) exhibited the highest scavenger activity in the DPPH free radical scavenging assay, with an IC_50_ of 17.25 µg/mL. Furthermore, compound **102** (Figure 59), demonstrating two electron-withdrawing chlorine atoms on the piperazine entity attached to the berberine ring, revealed remarkable radical scavenging potential in the DPPH assay, with an IC_50_ of 19.80 ± 0.94 µM, comparable to that of control drug ascorbic acid at 61.03 ± 0.66 µM and far better than berberine at 89.18 ± 1.60 µM. Compound **103** (Figure 59), with a naphthyl piperazine substituent, demonstrated the notable potency in the ABTS^•+^ analysis, with an IC_50_ value of 8.94 µM while the parent scaffold berberine with IC_50_ = 232.1 ± 2.11 µM and ascorbic acid with IC_50_ = 27.02 ± 0.05 µM. Compound **50** (Figure 25) showed significant antioxidant activity with an IC_50_ value of 11.08 and 4.76 µg/mL in DPPH and ABTS bioassays, respectively, while the parent scaffold berberine with IC_50_ = 34.29 µg/mL and 82.17 µg/mL and ascorbic acid with IC_50_ = 10.75 µg/mL and 5.528 µg/mL in the DPPH and ABTS bioassays, respectively^72^^,^^74–76^.

**Figure 60.**
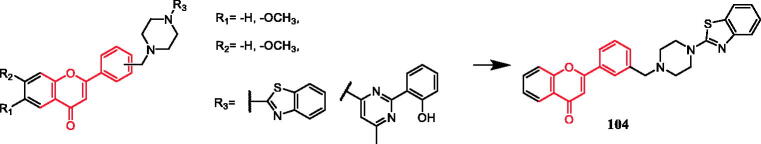
Chemical structures of piperazinyl flavone derivatives.

A series of 2-(2-hydroxyphenyl) pyrimidine/benzothiazole piperazinyl-substituted flavones were synthesised and evaluated for their in vivo biological properties. The results indicated that all tested compounds containing flavonoid-piperazine moieties were found to possess free radical scavenging potential. The SAR study showed that the antioxidant activity was decreased with methoxy group substitution on the flavone unit. Furthermore, the analogue **104** (Figure 60) benzothiazole substituted piperazine ring showed better antioxidant activity. Hydroxyl free radical scavenging capacity of analogue **104** at 1.25 mM (scavenging ratio = 48.0%) was comparable to positive control thiourea (scavenging ratio = 46.0%), and higher than α-tocopherol (scavenging ratio = 18.7%)^132^.

### 2.5. Nervous system activity

**Figure 61.**
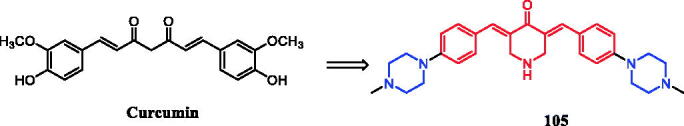
Chemical structures of curcumin and its derivative.

In 2011, by introducing N-methylpiperazine on aromatic compounds, it was expected that the hydrogen bond substitution and additional electrostatic interaction would significantly enhance the binding affinity with the A*β* peptide. Curcumin analog **105** (Figure 61) containing a piperazine ring reportedly demonstrated the most significant activity, with an IC_50_ value of 2.5 ± 1.2 µM and 90.2 ± 3.2% inhibition of A*β* aggregation at a concentration of 50 µM, a much higher activity than curcumin (IC_50_ value of 12.1 ± 1.2 µM and 71.3 ± 1. 3% inhibition of A*β* aggregation at a concentration of 50 µM)^133^.

**Figure 62.**
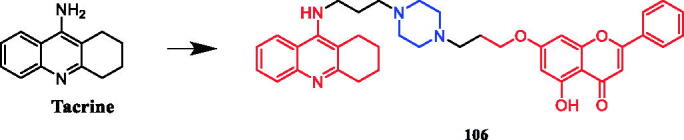
Chemical structures of tacrine and its derivative.

In 2013, tacrine-flavonoid hybrids were connected via piperazine side-arm alkyl groups. Among the series of compounds, compound **106** (Figure 62) displayed balanced inhibitory effects against acetylcholinesterase (AChE) and self-induced A*β*_1_*_−_*_42_ aggregation (IC_50_ = 133 nM for AChE, IC_50_ = 6.5 µM for aggregation), better than tacrine (IC_50_ = 260 ± 8 nM for AChE) and curcumin (IC_50_ = 20.3 ± 1.2 µM for aggregation). Furthermore, it exhibited moderate metal chelating activity and low cell toxicity. A molecular docking study indicated that the piperazine in the structure could bind to the AChE binding site in the middle of the canyon through a cation-*π* interaction (5.45 Å) between its protonated nitrogen atom and Tyr334 in the middle of the canyon^134^.

**Figure 63.**
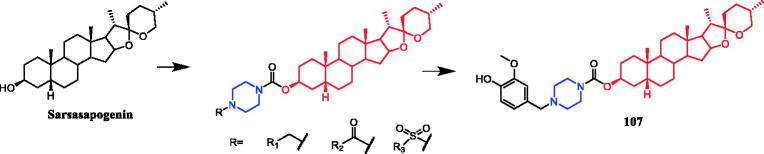
Chemical structures of sarsasapogenin and its derivatives.

In 2018, a series of multifunctional 3-piperazinecarboxylate sarsasapogenin derivatives were designed and synthesised against AD. The protective effect of compound **107** (Figure 63) at a concentration of 10 µM (Cell viability = 43.9 ± 2.8%) against the oxidative stress triggered by H_2_O_2_ in PC12 cells was stronger than that of positive control N-acetyl-L-cysteine at 1 mM (Cell viability = 35.3 ± 2.5%). Further SAR analysis indicated that benzyl, electron-donating groups, and intramolecular hydrogen bonding may help increase its neuroprotective activity. The report indicated that N-substituted piperazine carboxylate can be used as a promising structural unit for designing anti-AD drugs. Significantly, compound **107** protected PC12 cells from Aβ-induced damage and improved learning and memory impairments in Aβ-injected mice with low-dose administration at 6 mg/kg^135^.

**Figure 64.**
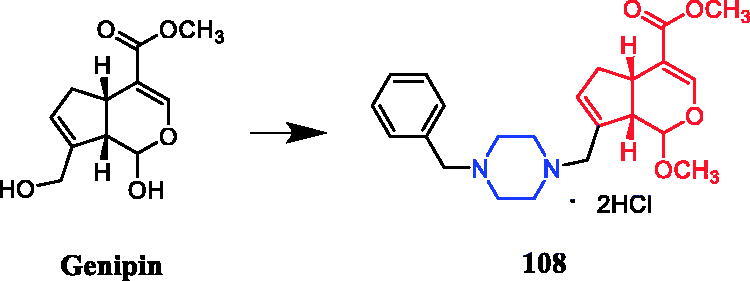
Chemical structures of genipin and its analog.

In 2019, genipin analog **108** (Figure 64) with a benzylpiperazine modification at C-8 position showed a higher inhibition rate (%Inhibition = 22.29) on protecting PC12 cells against injured by Aβ_1_–_42_ than the positive reference donepezil (%Inhibition = 17.65). For compounds containing methyl substituents, replacement at 3- position of the terminal benzene ring was more beneficial to the enzymatic activity than at 4-position substitution. In addition, the methoxy substitution at the terminal phenyl moiety was more advantageous than the methyl group. When an electron-withdrawing groups were introduced at the end of the phenyl ring, the increase in activity was consistent with the enhancement of the electron-withdrawing performance of the substituent. In the entire series, genipin derivatives with ligustrazine as the terminal aryl group were identified as the two most effective compounds. In terms of neuroprotective activity, compounds with benzene as the terminal aryl group revealed better neuroprotective effects than those with ligustrazine as the terminal aryl group^136^.

However, in 2020, Yang et al. found that the introduction of piperazine reduced the anti-AD effect of diosgenin carbamate derivatives^137^.

**Figure 65.**
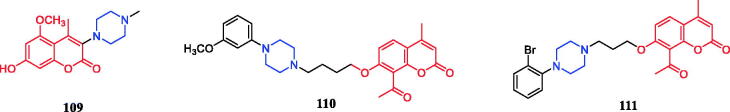
Chemical structures of coumarin and its derivatives.

Coumarin derivative **109** (Figure 65) demonstrated clear neuroprotection in the middle cerebral artery occlusion rat model by reducing the infarct size and brain-water content, improving neurological function, and suppressing neuronal loss and neuropathological changes in the cortex and hippocampus. Compound **109** at doses of 3.0 and 6.0 mg/kg significantly reduced the percentage of the infarct area within the ipsilateral hemispheres compared with the vehicle control group (20.36 ± 7.9% and 18.89 ± 5.83%, respectively). The same result was observed for Edaravone at 6.0 mg/kg, with which a significant reduction of brain-water content also occurred. Further pharmacokinetic studies have shown that compound **109** could penetrate the blood-brain barrier of rats^138^. Ostrowska and co-workers designed 7-hydroxy-4-methyl coumarin derivatives to increase the affinity to 5-hydroxytryptamine (5-HT) receptors. Compound **110 and 111** (Figure 65) showed higher affinities to 5-HT_1A_ receptors (*K*_i_ value of 0.8 ± 0.009 and 0.50 ± 0.05 nM, respectively) than serotonin (1.3 ± 0.1 nM). Reportedly, substituents at ortho or meta positions in the phenyl group of piperazine play a decisive role in the affinity to 5-HT_1A_ receptors^139^^,^^140^.

**Figure 66.**
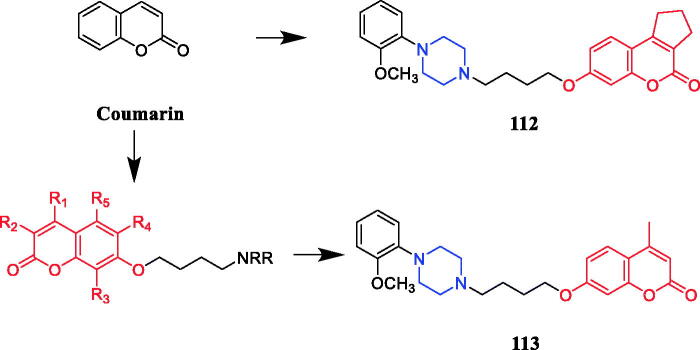
Chemical structures of coumarin and its derivatives.

The synthesis and evaluation of novel, potential antipsychotic coumarin derivatives were undertaken by Yin Chen et al. Compound **112** (Figure 66) displayed a high affinity for 5-HT_1_*_A_*, 5-HT_2_*_A_*, and dopamine (D_2_) receptors (*K*_i_ = 0.2, 3.9 and 5.6 nM, respectively). Based on the results, compound **112** exhibited a higher affinity for the 5-HT_1A_ receptor when compared with risperidone (K*_i_* = 190.2 nM). Moreover, compound **112** demonstrated lower affinity for H_1_ receptors (*K*_i_ = 699.1 nM) when compared with risperidone (*K*_i_ = 22.9 nM) and clozapine (*K*_i_ = 6.8 nM)^141^. Chen et al. reported a series of coumarin piperazine (piperidine) derivatives as potential multireceptor atypical antipsychotics. The SAR has shown that several factors affected the binding affinity for the D2, 5-HT_1_A, and 5-HT_2_A receptors of these compounds. (1) Bearing a 6-fluorobenzo[d]isoxazol-3- yl)piperidine moiety exhibited higher affinities to three receptors than those with other amine moieties, (2) the substitution on the 4-position (R_1_) with methyl as the favoured substituent, (3) the substitution on the 3-position (R_2_) with unsubstituted or substitution (methyl) preferred, and (4) the substitution on the 8-position (R_3_) with electron-withdrawing (chloro) as the preferred substituent; (5) the substitution on the 5-position (R_5_) or 6-position (R_4_) did lower the affinity for the three receptors, (6) the importance of the double bond for affinity to D2, 5-HT_1_A and 5-HT_2_A receptors, and (7) straight four-carbon chain alkyl was superior to another alkyl linker. Notably, piperazine derivative **113** (Figure 66) was considered among the most promising derivatives investigated, displaying the strongest affinity for 5-HT_1_*_A_* receptors. According to the results, compound **113** exhibited a higher affinity for D_2_ and 5-HT_1_*_A_* receptors (D_2_, *K*_i_ =1.4 nM; 5-HT_1A_, *K*_i_ = 0.012 nM) when compared with risperidone (D_2_, *K*_i_ = 3.7 nM; 5-HT_1A_, *K*_i_ = 180 nM)^142^.

**Figure 67.**
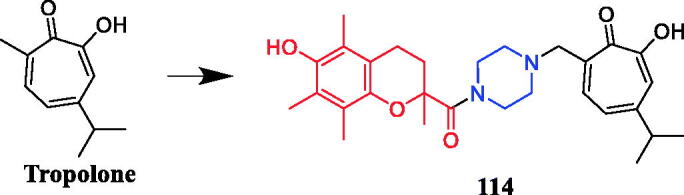
Chemical structures of tropolone and its derivative.

Generally, the introduction of the piperazine group improved the neuroprotective activity when compared with tropolone according to a study reported by Koufaki et al. Compound **114** (Figure 67) showed the most potent protective activity against glutamate-challenged HT22 cells, with an EC_50_ value of 0.08 ± 0.02 µM (the EC_50_ value of positive control drug tropolone >10 µM, β-thujaplicin = 4.80 ± 0.42 µM)^143^.

**Figure 68.**
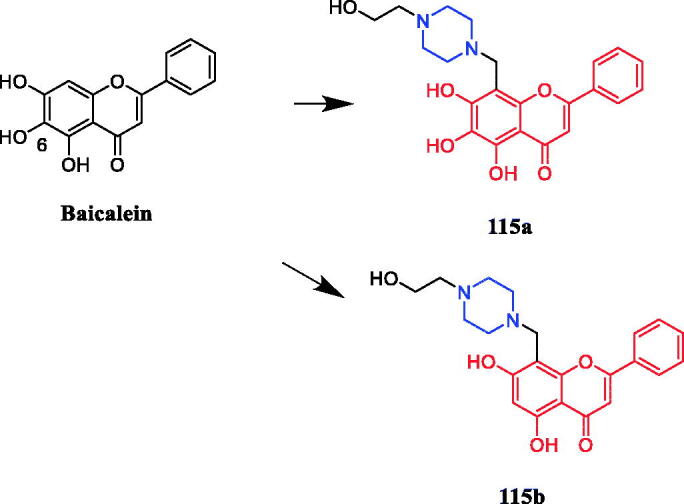
Chemical structures of baicalein and its derivative.

A series of flavone derivatives were synthesised and evaluated for their H_3_R inhibitory activity. Among them, a compound containing piperazine ring **115a** (Figure 68) exhibited good inhibitory activity against H_3_R. The H_3_R inhibition rate treated with compound **115a** at doses of 25 µmol/ml was 81.32 ± 4.3% while positive control thiazole amide at 5 µmol/mL was 60.92 ± 3.5%. The results revealed that the hydroxyl at C6 also showed a significant effect by comparing compound **115a** with **115 b (**H_3_R inhibition rate = 12.31 ± 4.5% at doses of 25 µmol/mL**)** (Figure 68)^144^.

**Figure 69.**
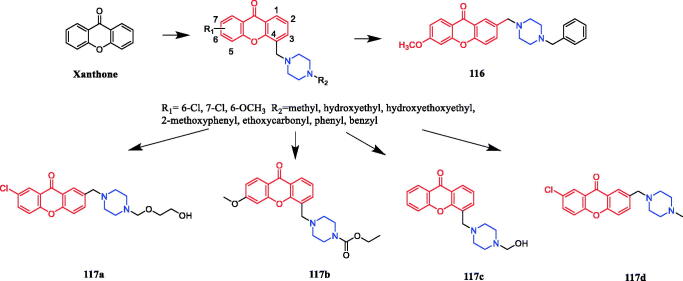
Chemical structures of xanthone and its derivatives.

Waszkielewicz et al. reported that a xanthone derivative with piperazine moiety **116** (Figure 69) showed excellent anticonvulsant activity, with an ED_50_ value of 105 mg/kg in the maximal electroshock (MES) seizure assay. In the anticonvulsant test, the mice were administered 0.5 or 4 h before the test, and the doses of the drug were 30, 100 and 300 mg/kg, respectively. Finally, derivative **116** proved to have better performance because it showed no neurotoxicity at both test time points, but both 100 mg/kg and 300 mg/kg were active in MES at 4 h after administration. In another study, xanthone derivative **117a**–**117d** (Figure 69) containing a piperazine moiety was evaluated for interactions with major classes of G-protein coupled receptors (GPCRs), uptake systems, and ion channels, revealing that it binds to 5-HT_2A_, 5-HT_2B_ receptors, and sodium channels. And all derivatives synthesised were assessed using the SwissADME platform, the results revealed most of the compounds fulfilled Lipinski’s rule of five along with good BBB (blood-brain barrier) penetration. In the for-plate test compound **117a**–**117d** significantly increased the number of punished crossings at dose 5 mg/kg by 66.6% and at the dose 10 mg/kg by 50.1%, respectively. Furthermore, derivative **117a**–**117d** were evaluated by measuring mean plasma and brain concentrations after intragastric administration in mice at a dose of 5 mg/kg. Among the molecules, compound **117b** had the longest half-life, ca. 3 h compared to compound **117a** (ca. 1 h). The compound **117d** showed the largest apparent volume of distribution (41.3 l/kg) that indicated the ability for penetration to the deep compartment and binding to tissues. After i.p. administration, all compounds quickly, within 15 min achieve maximum concentration (*C*_max_) in the blood. The molecules were characterised by a high permeability to the brain, with the highest brain to plasma ratio (31.6) for compound **117d**. As for the SAR, the partial substitution of piperazine at the 4 positions of the heteroanthrone structure is more conducive to enhancing the antidepressant and anti-anxiety activities in vivo than the 2 positions. Besides, the presence of methoxy or chloro substituent at 6 or 7 positions had a favourable effect on the antidepressant activity and anti-anxiety activity^145^^,^^146^.

### 2.6. Cardiovascular activity

**Figure 70.**
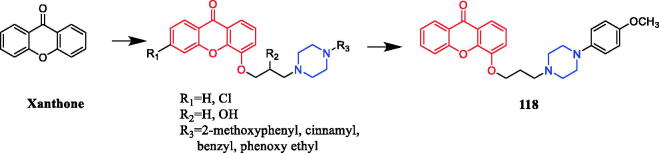
Chemical structures of xanthone and its derivatives.

A series of xanthone derivatives with piperazine were synthesised and evaluated for their cardiovascular activity. Compound **118** (Figure 70) exhibited significant antiarrhythmic activity, with an ED_50_ value of 0.69 mg/kg in adrenaline-induced arrhythmia, while ED_50_ values of Propranolol, Urapidil, Carvedilol were 1.05, 1.26, 0.25 mg/kg, respectively. From the structure-activity relationship, it can be considered that the most active compound contained a 2-methoxyphenylpiperazine moiety. Compounds containing 4-methoxyphenylpiperazine moieties are less effective in *in vivo* tests than their 2-substituted analogs. In vivo experiments showed that the binding affinity of all test compounds to the α_2_-receptors and β_1_-adrenoceptors were within the µM-range, and revealed the structural relationship between them. The introduction of chlorine substituents on the xanthone ring also reduced the binding affinity of the α_1_-adrenergic receptors, indicating that the increase in lipophilicity is undesirable^147^.

**Figure 71.**
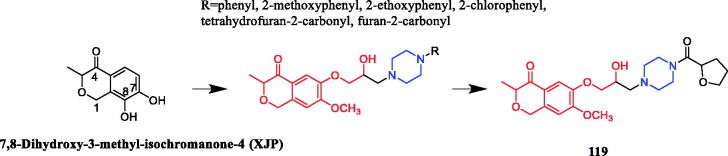
Chemical structures of 7,8-dihydroxy-3-methyl-isochromanone-4 (XJP) and its derivatives.

In a series of polyphenolic natural product analogs bearing an arylpiperazine moiety developed by Xie et al., compound **119** (Figure 71) acted as a potential antihypertensive agent in spontaneously hypertensive rats (SHRs). In biological investigations, compound **119** reduced the systolic and diastolic blood pressure in SHRs comparable to naftopidil. The studies have shown that the mean arterial pressure (MAP) of **119** on SHR was reduced by nearly 23.7% at 4 h, which is better than the 16.1% of naftopidil at 2 h, demonstrating compound **119** may possess no effects on the basal heart rate. The SAR analysis showed that the substitution position of the linker had an effect on vasodilation activity. In general, most analogs with C6 substitution are more effective than corresponding analogues with C7 and C8 substitution^148^.

### 2.7. Other biological activity

**Figure 72.**
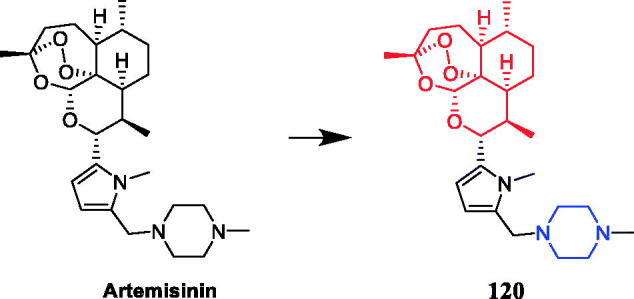
Chemical structures of artemisinin and its derivatives.

16 semisynthetic C-10 pyrrole Mannich artemisinin derivatives were first evaluated *in vitro* against the chloroquine-sensitive 3D7 strain of P. falciparum, several of which are more than three times more effective than the natural product artemisinin. According to the analysis of the optimal Mannich side-chain substitution for *in vitro* and in vivo activity, the morpholine and N-methylpiperazine in the Mannich side-chain substitution **120** (Figure 72) displayed the best activity among these derivatives. According to the SAR analysis, morpholine, N-methylpiperazine, and sulfonylmorpholine heterocyclics could provide molecules with increased potency both *in vitro* and *in vivo*. Furthermore, compound **120** had significantly better antimalarial activity in vivo versus the *P. Berghei N strain* (ED_50_ = 1.99 mg/kg, ED_90_ = 5.34 mg/kg, respectively) than water-soluble sodium artesunate (ED_50_ = 3.23 mg/kg, ED_90_ > 10 mg/kg, respectively)^149^.

**Figure 73.**
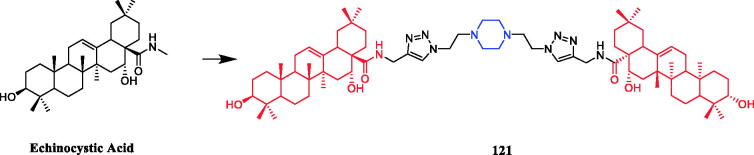
Chemical structures of echinocystic acid and its derivatives.

To improve solubility and maintain some rigidity, pentacyclic triterpene echinocystic acid dimer **121** (Figure 73) was designed and synthesised by replacing the phenyl group of the linker with piperazine, demonstrating significant anti-hepatitis C (HCV) entry activity, with an IC_50_ value of 2.87 nmol/L, two orders of magnitudes more potent than EA (1.4 µmol/L). In addition, the haemolysis of the synthesised compound is eliminated under the concentration gradient of 0, 6.25, 12.5, 25 and 50 mmol/L, while EA had obvious haemolytic side effects when the CC_50_ was 15 mmol/L^150^.

**Figure 74.**
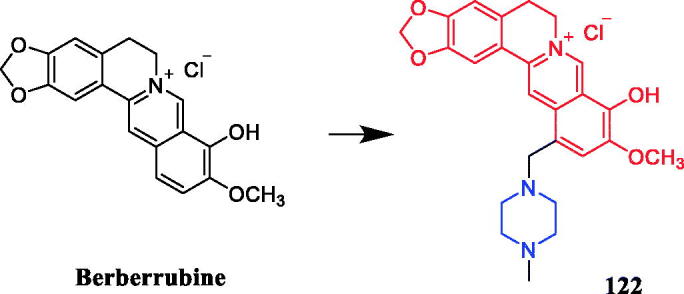
Chemical structures of berberrubine and its derivative.

Li et al. synthesised a series of berberrubine derivatives by introducing various aminomethyl groups into 12-position of berberrubine and evaluated their anti-diabetic activity against type 2 diabetes mellitus. Compound **122** (Figure 74) with an N-methyl piperazine-4-methyl group at C-12 showed the most potent anti-diabetic activity and the sensitisation reached 1.26 fold of rosiglitazone. Even if the concentration dropped to 1 µmol/ml, its activity was still comparable to the positive control rosiglitazone. The research suggested compound **122** had a certain effect on promoting glucose uptake on insulin-resistant 3T3-L1 adipocytes and L6 myotubes^151^.

**Figure 75.**
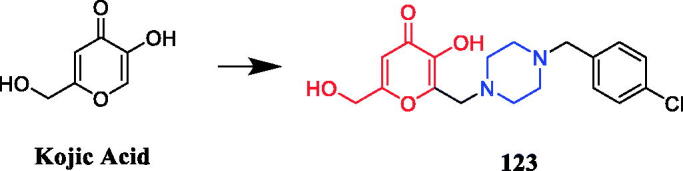
Chemical structures of kojic acid and its derivative.

Based on the relationship between results of tyrosinase inhibition and molecular docking, kojic acid derivative **123** (Figure 75) was found to be a promising antityrosinase agent, and the hydroxymethyl group in the structure provided a metal complex of copper ions and chlorine atoms, allowing conventional hydrogen bonds to interact with tyrosinase, with an IC_50_ value of 86.2 µM. Compound **123** showed the antidiphenolase activity of mushroom tyrosinase to be almost 5-fold higher than that of control agent kojic acid (IC_50_ = 418.2 µM)^152^.

**Figure 76.**
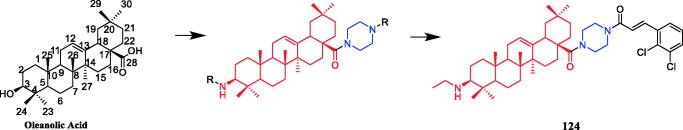
Chemical structures of oleanolic acid and its derivatives.

Nie et al. synthesised a series of *α*-glucosidase inhibitors based on OA. The piperazine fragment was introduced to link the cinnamic amide unit with OA at C-28. Compound **124** (Figure 76) with 3, 28-disubstituted OA exhibited potent inhibitory activity, with an IC_50_ value of 1.90 µM. Interestingly, the different substituents on the benzene ring of the cinnamic amide unit had a significant effect on the activity. For example, fluorine atoms can reduce the activity, and the expansion of the hydrophobic surface could also enhance the inhibitory activity of α-glucosidase. However, compared with oleanolic acid (IC_50_ = 98.5 mM), the introduction of cinnamamide at position C-28 did not result in a significant increase in α-glucosidase inhibitory activity. The activity of 3-OH derivatives had stronger effects than that of 3-OAc derivatives, possibly due to its good steric binding to enzymes. The 3,28-disubstituted oleanolic acid derivatives significantly improved the inhibitory activity of α-glucosidase than oleanolic acid^153^.

**Figure 77.**
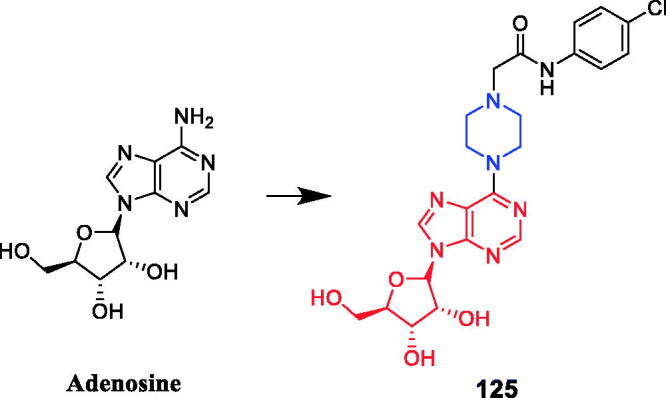
Chemical structures of adenosine and its analog.

Reportedly, adenosine analog with piperazine **125** (Figure 77) exhibited better inhibitory activity against Coxsackievirus B3 (CVB3), with a lower IC_50_ value of 5.1 ± 2.3 mg/L and a higher selectivity index (TI = 41.0) than ribavirin (IC_50_ = 36.8 ± 9.6 mg/L, TI = 29.1, TI, selectivity index represented by the TC_50_/IC_50_ ratio)). The studies have shown that in this series of compounds, the phenyl substitution of piperazine moiety may be more effective than pyridine substitution to enhance antiviral activity. This may be due to the lone pair of electrons in the aromatic ring. In addition, the calculated log *p* values of **125** were 0.8 and met the log P criteria, and the acute toxicity level was within 50 ≤ LD_50_ < 500 mg/kg, with no carcinogenic and mutagenic toxicity risk^154^.

## 3. Conclusion

Piperazine is a part of numerous natural and synthetic molecules, with broad therapeutic potential. In addition to being a pharmacophore, piperazine is used as a linker/bridge between natural products and active structural molecules. This review summarises the research progress in the synthesis of natural product-piperazine derivatives in the past ten years, aiming to discover natural product piperazine hybrid compounds that may possess abundant biological activities. This will aid the scientific community in rationally design and develop novel, targeted, optimised, and diversified natural products-piperazine drugs for the treatment of multifactorial diseases. In conclusion, the piperazine group is widely used in for drug synthesis, attracting great attention from researchers worldwide, and its role cannot be ignored.
